# Molecularly Engineered Fluorescent Magnetic Microrobots for Sensing High‐Energy Nitroaromatic Explosives in Highly Acidic Aqueous Environments

**DOI:** 10.1002/smll.202512670

**Published:** 2025-12-09

**Authors:** Nikhil Thekkedath Madhu, N. Senthilnathan, Martin Pumera

**Affiliations:** ^1^ Future Energy and Innovation Laboratory Central European Institute of Technology Brno University of Technology Purkynova 123 Brno 61200 Czech Republic; ^2^ Advanced Nanorobots & Multiscale Robotics Laboratory Faculty of Electrical Engineering and Computer Science VSB – Technical University of Ostrava 17. listopadu 2172/15 Ostrava 70800 Czech Republic; ^3^ Department of Medical Research China Medical University Hospital China Medical University No. 91 Hsueh‐Shih Road Taichung 40402 Taiwan; ^4^ Department of Chemical and Biomolecular Engineering Yonsei University 50 Yonsei‐ro, Seodaemun‐gu Seoul 03722 South Korea

**Keywords:** explosives, microfluidics, micromotors, nanorobots

## Abstract

Among various analytical sensing approaches currently in use, fluorescence sensing is known for its high sensitivity, rapid response, and applicability in monitoring a wide range of target molecules. Pairing an appropriate navigation system with a fluorescent molecule that can carry it to inaccessible or hard‐to‐reach environments can pave the way for remote and selective sensing applications. Herein, we design magnetic nanoparticles embedded with fluorescent material to form magneto‐fluorescent, precisely navigable microrobots for sensing high‐energy explosive compounds in acidic aqueous systems. Magnetic guidance via Helmholtz coils provides dynamic navigational control to direct the microrobots to specific regions of interest using externally applied magnetic fields. Upon specific interaction with picric acid, the sensing mechanism is triggered via fluorescence quenching (“on‐off” switch). Materials characterization demonstrates that this quenching mechanism arises from the hydrogen bonding and charge‐transfer interactions between ketenimine‐functionalized probes and the hydroxyl group in picric acid, leading to the formation of water‐soluble picrate complexes. Testing in microfluidic channels as proof‐of‐concept further validates the microrobot’s ability for selective sensing of target analytes, emphasizing them as smart mobile sensors for environmental monitoring in harsh acidic conditions, confined spaces, hazardous material detection, and applications in the real world where conventional sensors face challenges.

## Graphical abstract

Magnetically powered fluorescent microrobots embedded with ketenimine‐based probes enable remote sensing of high‐energy explosive compounds in harsh acidic environments. Upon interaction with picric acid, the fluorescence of the microrobots is quenched, providing a precise “on–off” detection mechanism for selective monitoring in confined and hazardous areas.

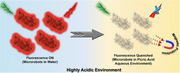

## 1 Introduction

With the growing threat of organized militant groups and terror attacks around the world, there is an urgent need for the detection of explosive compounds that are more sophisticated, reliable, and easy to use.^[^ ^1^, ^2^, ^3^, ^4^ ^]^ Nitroaromatic compounds are the main components in many explosives.^[^ ^5^, ^6^ ^]^ Among these explosives, 2,4,6‐trinitrophenol, also known as picric acid, stands out as especially dangerous because of its greater explosive power, strong acidity, and the presence of three nitro groups that make it super reactive and explosive.^[^ ^7^, ^8^ ^]^ Besides being used in weapons and rocket fuels, picric acid is also employed in various industrial areas, such as pharmaceuticals, agrochemicals, leather processing, and fireworks.^[^ ^9^, ^10^ ^]^ Even tiny traces of picric acid can be harmful, causing skin and eye burns, breathing problems, and long‐term health issues such as anemia, cancer, bluish skin (cyanosis), and liver and kidney damage.^[^ ^11^, ^12^ ^]^ Because picric acid has a molecular structure similar to other nitroaromatic chemicals, detecting it specifically can be quite tricky, so we need sensitive and selective tools for sensing.^[^ ^13^, ^14^ ^]^ Several platforms, like metal–organic frameworks,^[^ ^15^ ^]^ conjugated polymers,^[^ ^16^ ^]^ dendrimers,^[^ ^17^ ^]^ quantum dots,^[^ ^18^, ^19^ ^]^ and nanoparticle probes,^[^ ^20^ ^]^ have shown promise. Lately, fluorescent small organic molecules are gaining popularity because they are safer, more eco‐friendly, and easier to modify structurally.^[^ ^21^, ^22^, ^23^ ^]^ In this work, our goal is to create new types of fluorescent small‐molecule sensors that can specifically spot picric acid over other nitroaromatic explosives in a highly acidic environment, which is a challenging task.

Currently, micro‐ and nanorobotic systems are emerging in various fields for their efficiency in moving through complex areas very accurately under external stimuli.^[^ ^24^, ^25^, ^26^, ^27^, ^28^, ^29^ ^]^ They can carry out programmable locomotion and operate with high adaptability in confined, dynamic, or unreachable areas.^[^ ^30^, ^31^ ^]^ Various external stimuli, including light irradiation,^[^ ^32^, ^33^, ^34^, ^35^ ^]^ magnetic field,^[^ ^36^, ^37^, ^38^ ^]^ electric field,^[^ ^39^, ^40^, ^41^ ^]^ and acoustic force, were used to locomote the micro/nanorobots.^[^ ^42^, ^43^ ^]^ Among them, magnetic field‐driven actuation has proven to be very attractive because of its non‐invasive and long‐range control.^[^ ^44^, ^45^, ^46^ ^]^ Subsequently, significant applications of magnetically responsive microrobots in biomedicine, environmental monitoring, and pollutant remediation have been studied.^[^ ^47^ ^]^ Different propulsion modalities and functionalizations are being explored to design microrobots that can perform specific tasks, such as localized drug delivery,^[^ ^48^ ^]^ biofilm disruption,^[^ ^49^ ^]^ and removal of bacteria^[^ ^50^ ^]^ and chemical contaminants in aqueous systems.^[^ ^51^ ^]^ Microrobotic systems have been receiving considerable attention for both the degradation and removal of pollutants.^[^ ^52^, ^53^, ^54^, ^55^ ^]^ However, relatively few studies have focused on the sensing of environmental toxins, such as nitroaromatic explosives, in acidic environments.^[^ ^56^ ^]^ This work emphasizes the development of a magnetically actuated fluorescent microrobot based on quinonoid structure for the specific sensing of picric acid in highly acidic, complex, and inaccessible aqueous environments. The microrobots are composed of a diaminodicyanoquinodimethane (DADQ) derivative that has strong fluorescence and responsive optical switching ability under environmental stimuli.^[^ ^57^, ^58^ ^]^ In this molecular design, precise functionalization is engineered for selective interaction with nitroaromatic compounds. With its high biocompatibility, these microrobots could be highly suitable for real‐time environmental monitoring.^[^ ^59^, ^60^ ^]^ In our group, we recently published a work on the detection of nitroaromatic explosives using DADQ derivative, which has the limitation of sensing in acidic conditions.^[^ ^61^ ^]^ The DADQ‐based microrobots fail to detect picric acid under acidic conditions due to material instability and fluorescence quenching in the acidic environment (pH 2–5). In contrast, microrobots developed in this work maintain strong fluorescence and sensing ability even at pH 2, enabling reliable detection in harsh acidic environments. However, this newly developed system represents a new class of promising intelligent microrobots for autonomous sensing applications in harsh acidic conditions.

**Scheme 1:**
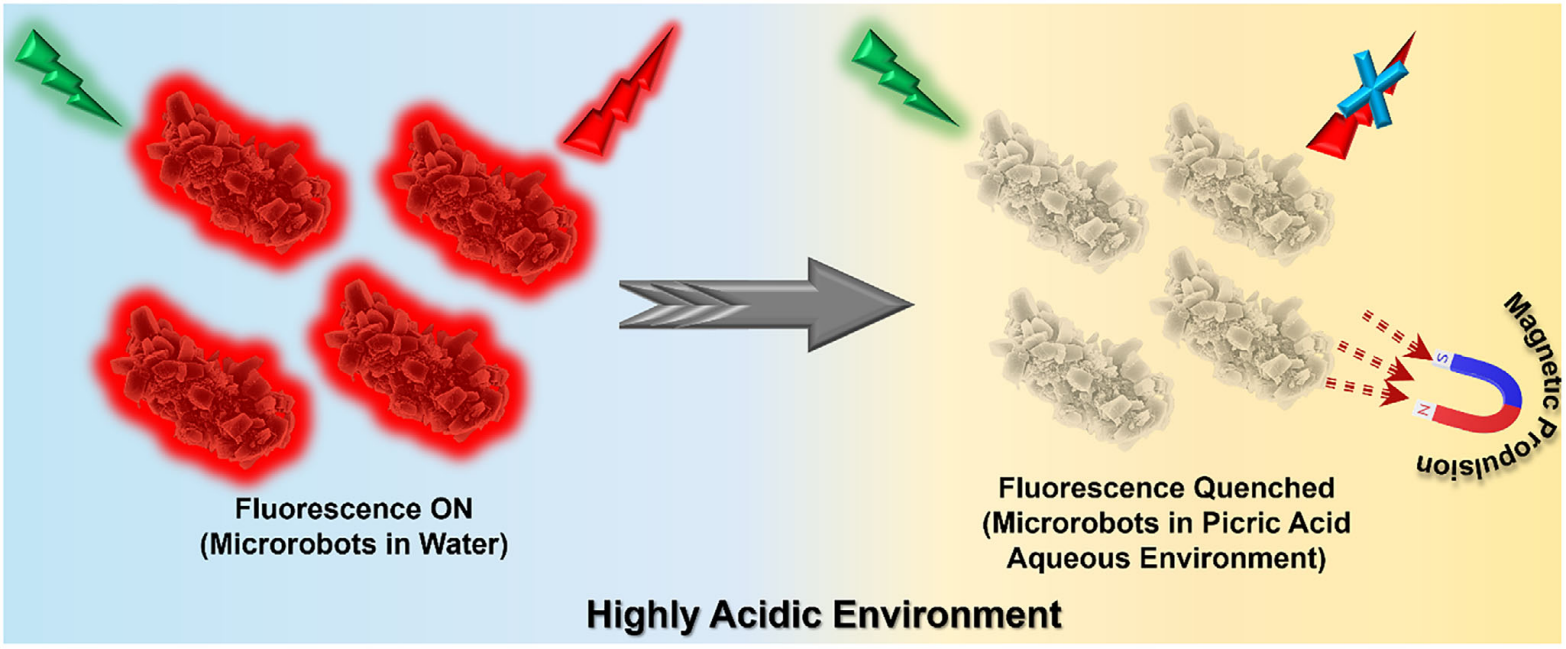
Magnetic propulsion of fluorescent microrobots for the sensing of picric acid in an acidic aqueous environment.

In this work, a hybrid microrobot is designed and developed for the selective sensing of picric acid in a highly acidic aqueous environment. During the fabrication, the core sensing material is a molecularly engineered fluorescent organic compound, 7,7‐bis(piperidino)‐8,8‐dicyanoquinodimethane – Green (BP‐G). Upon protonation with trifluoroacetic acid (TFA), BP‐G turns from green to deep red fluorescence, forming a ketenimine‐based form known as 7,7‐bis(piperidino)‐8,8‐dicyanoquinodimethane – Red (BP‐R). Thus, the system can be custom‐tuned and works well with the adaptable and selective sensing based on fluorescence. Integration of Fe_3_O_4_ magnetic nanoparticles into the matrix of the microrobots offers magnetic responsiveness for remote manipulation, guided locomotion, and easy retrieval by external magnetic fields. The design's dual functionality, such as optical sensing and magnetic actuation, allows the microrobots to adaptively perform real‐time sensing in complex fluidic environments. The functional performance of the microrobots in real‐world scenarios, such as inaccessible or hard‐to‐reach environments, like industrial effluent streams, chemical waste pipelines, storage tanks, and contaminated groundwater channels, was validated by sensing picric acid in acidic conditions and using fluidic narrow channels. The main advantage of the BP‐R microrobots is the capability to recover the microrobots after detection of selective explosives, which prevents secondary contamination. The fluidic setup serves as a proof‐of‐concept platform and representative model for demonstrating targeted navigation, transport, and sensing in hard‐to‐reach aqueous environments. The interaction of microrobots with picric acid has been systematically characterized by spectroscopic and microscopic techniques to evaluate the structural and optical responses, which show remarkable fluorescence switching behavior. By synergizing selective molecular recognition, switchable fluorescence, and magnetically guided mobility, this study provides a novel and modular platform for intelligent environmental monitoring even in acidic conditions. This strategy promises to bring new avenues into the manufacturing of smart microdevices capable of performing on‐site sensing of hazardous materials for real‐time application (**Scheme** **1**).

## 2 Results and Discussion

### 2.1 Synthesis, Fabrication, and Characterization

**Figure 1:**
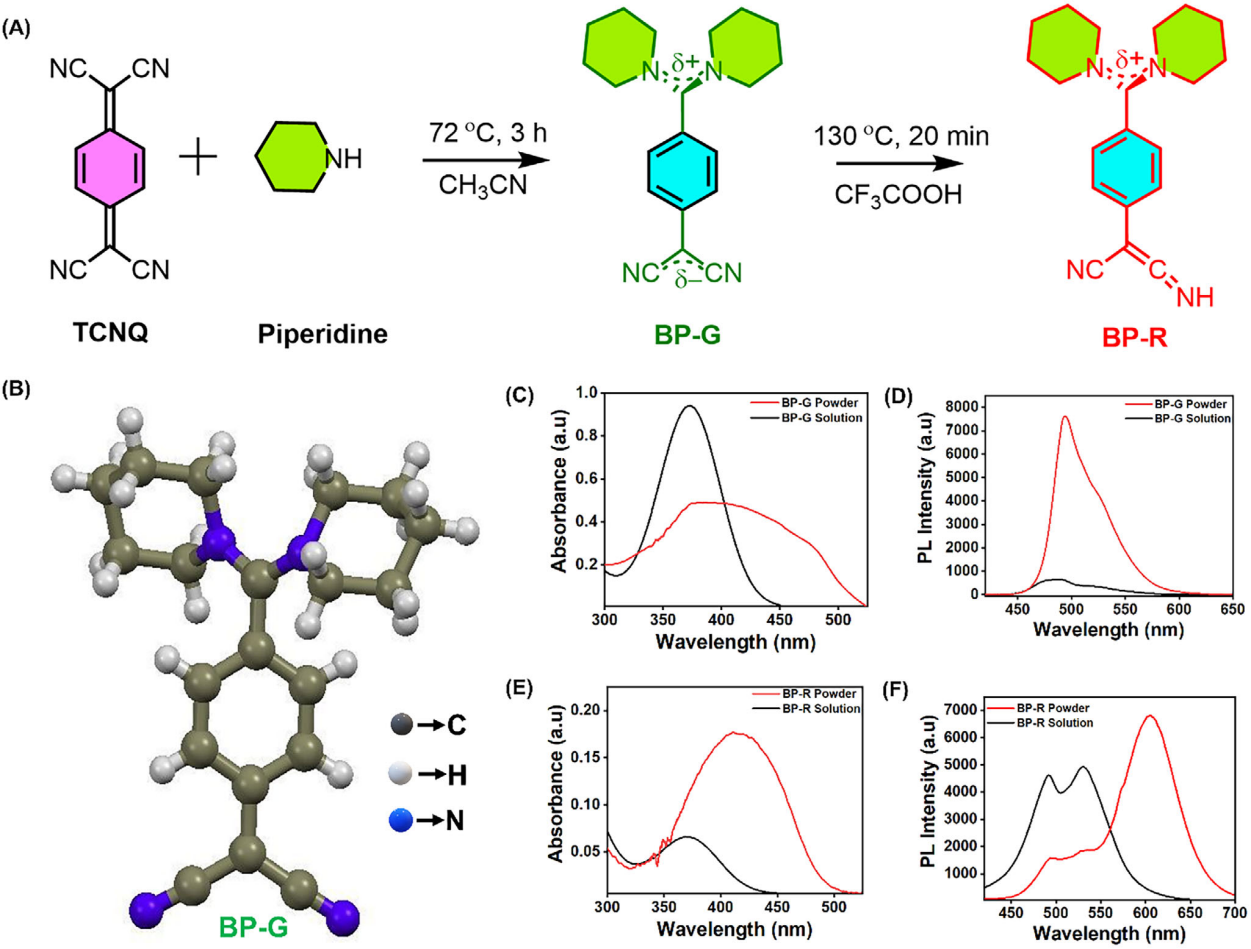
Synthesis and characterization of 7,7‐bis(piperidino)‐8,8‐dicyanoquinodimethane BP‐G and BP‐R. A) Scheme for the synthesis of BP‐G and BP‐R. B) Crystal structure of BP‐G molecule determined by single‐crystal X‐ray diffraction analysis, C) absorbance spectra, and D) photoluminescence emission spectra of BP‐G molecules in solid and solution states. E) Absorbance spectra and F) photoluminescence emission spectra of BP‐R molecules in solid and solution states.

BP‐R was synthesized via a two‐step procedure, wherein the BP‐G was first synthesized as shown in **Figure** **1**A. By acid‐assisted thermal treatment using trifluoroacetic acid, BP‐G was converted into its ketenimine version, BP‐R. Structural properties of BP‐G were studied using single‐crystal X‐ray diffraction (SCXRD, Figure 1B) and nuclear magnetic resonance (NMR) spectrometric analysis (Figure S1, Supporting Information). This analysis confirmed that a well‐ordered crystalline phase was produced. The single crystals of BP‐G formed from the acetonitrile solution, having a triclinic crystal structure with symmetry group *P ‐1*. The necessary crystallographic information is summarized in Table S1 (Supporting Information). Optical properties of BP‐G and BP‐R were studied both in the powder and solution forms. The spectra of UV‐visible absorption for BP‐G are shown in Figure 1C. In the solid state, it shows an absorption band at ≈380 nm while in the solution, it appears at 370 nm. It is observed that the optical properties of BP‐G are changed significantly after interacting with trifluoroacetic acid, as depicted in Figure 1E. Their absorption maximum is exhibited at 410 and 370 nm in their solid and solution states, respectively. The exhibited redshift in absorption spectra from ≈370 nm in solution to 410 nm in the solid state indicates that in the solid phase, there are enhanced intermolecular π–π interactions and molecular packing effects that stabilize the excited state and reduce the energy gap for lower‐energy (longer‐wavelength) absorption.^[^ ^62^ ^]^ As shown in Figure 1D, BP‐G emits very weakly in solution at 485 nm while in the solid state it has an intense peak at ≈495 nm. As depicted in Figure 1F, in the solid state, BP‐R exhibits bright fluorescence emission at ≈625 nm while in solution the emission peak decreases and shifts to ≈530 nm. The observed redshift in the emission of BP‐R from 530 nm in solution to 625 nm in the solid state is mainly due to enhanced intermolecular π–π interactions, aggregation effects, and restricted molecular motion in the solid phase, which stabilize the excited state, lower the energy gap, and thereby lead to emission at a longer wavelength.

**Figure 2:**
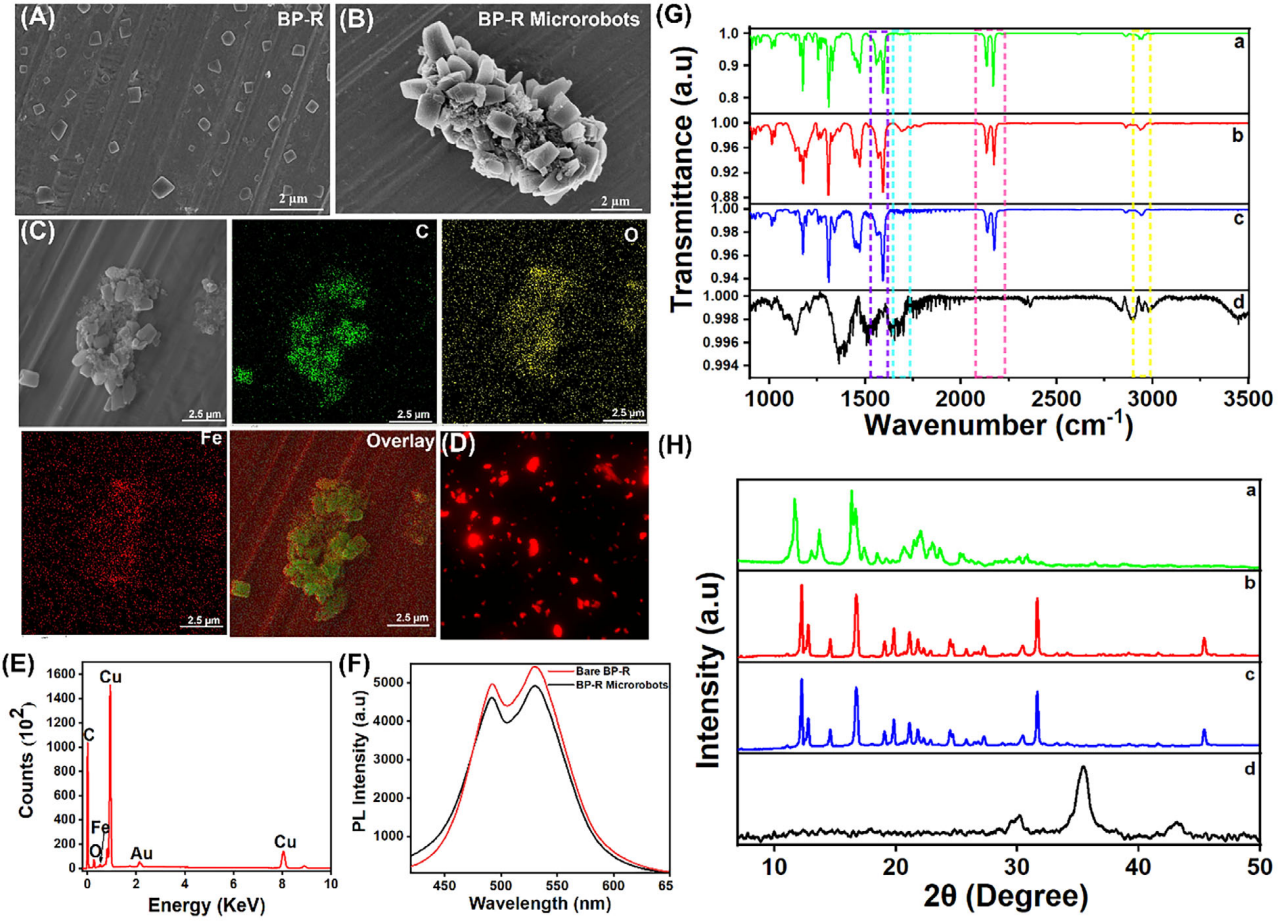
Characterizations of BP‐R microrobots. Scanning electron microscopy images of A) BP‐R microparticles and B) BP‐R microrobots (scale bars: 2 µm). C) Energy‐dispersive X‐ray spectroscopy elemental mapping of a single BP‐R microrobot showing C, O, Fe, and the overlay (scale bars: 2.5 µm). D) Fluorescence micrograph of BP‐R microrobots. E) Elemental mapping of single BP‐R microrobots. F) Photoluminescence emission spectra of BP‐R microparticles and BP‐R microrobots. G) Fourier‐transform infrared spectroscopy spectra and H) powder X‐ray diffraction spectra of (a) BP‐G, (b) BP‐R, (c) BP‐R microrobots, and (d) Fe_3_O_4_ nanoparticles.

Magnetic responsiveness was imparted by a straightforward reprecipitation process by decorating BP‐R microparticles with Fe_3_O_4_ nanoparticles. With this incorporation of iron oxide nanoparticles, BP‐R microparticles were magnetized for navigation under external magnetic fields. Scanning electron microscopy (SEM) results of the obtained microrobots showed a size in the range of 4–10 micrometers. Moreover, it is observed that the morphology of BP‐R microrobots is significantly varied from that of BP‐R microparticles, as shown in **Figures** **2**A,B. The incorporation of Fe_3_O_4_ nanoparticles during the fabrication process causes agglomeration, which leads to the formation of a new hybrid architecture with improved magnetic properties. Energy‐dispersive X‐ray spectroscopy (EDS) analysis, as shown in Figures 2C,E, indicates that the microrobots are mainly composed of carbon, confirming that the core structure comprises BP‐R microparticles rich in carbon. A significant presence of iron and oxygen indicates successful surface functionalization with Fe_3_O_4_ nanoparticles. The major Fe and O peaks corroborate the strong linking of the magnetic nanoparticles. Red‐colored emission are observed from BP‐R microrobots as shown in their fluorescence microscopic analysis in Figure 2D, and it is corroborated by their recorded fluorescence emission spectrum. As indicated in Figure 2F, it has been observed that functionalization with Fe_3_O_4_ nanoparticles slightly decreases the emission intensity of the bare BP‐R microparticles, reflecting a slight disruption of their optical properties. This suggests that the process of magnetic functionalization does not interfere significantly with their intrinsic optical properties. Fourier‐transform infrared spectroscopy (FTIR) and powder X‐ray diffraction (PXRD) spectra were analyzed to investigate the chemical and structural integrity of BP‐R microrobots. In the FTIR spectra, as shown in Figure 2G, it is seen that the characteristic peaks of BP‐R microrobots strongly align with those of bare BP‐R microparticles and BP‐G, thereby confirming their intact molecular structure during fabrication. Peaks at 2941 cm^−1^ (N─H stretching), 2172 and 2135 cm^−1^ (conjugated nitriles), and 1595 cm^−1^ (C═C stretching of the aromatic ring), indicative of the structural motifs of BP‐R compounds. Formation of a new peak at 1680 cm^−1^ in BP‐R shows protonation of the negatively charged cyano group, resulting in a ketenimine (═C═NH) moiety.^[^ ^58^ ^]^ PXRD analysis shown in Figure 2H further complements the above findings in demonstrating that the diffraction pattern of BP‐R microrobots closely resembles that of bare BP‐R microparticles, underscoring the retention of their crystalline phase post‐fabrication. In contrast, the PXRD pattern of BP‐G distinctly differs from that of BP‐R microrobots in crystalline structure. This set of results, therefore, demonstrates that the crystallinity of BP‐R compounds was not altered during the fabrication process, ensuring their continued functional usefulness in microrobotic applications.

### 2.2 Magnetic Actuation Studies

**Figure 3:**
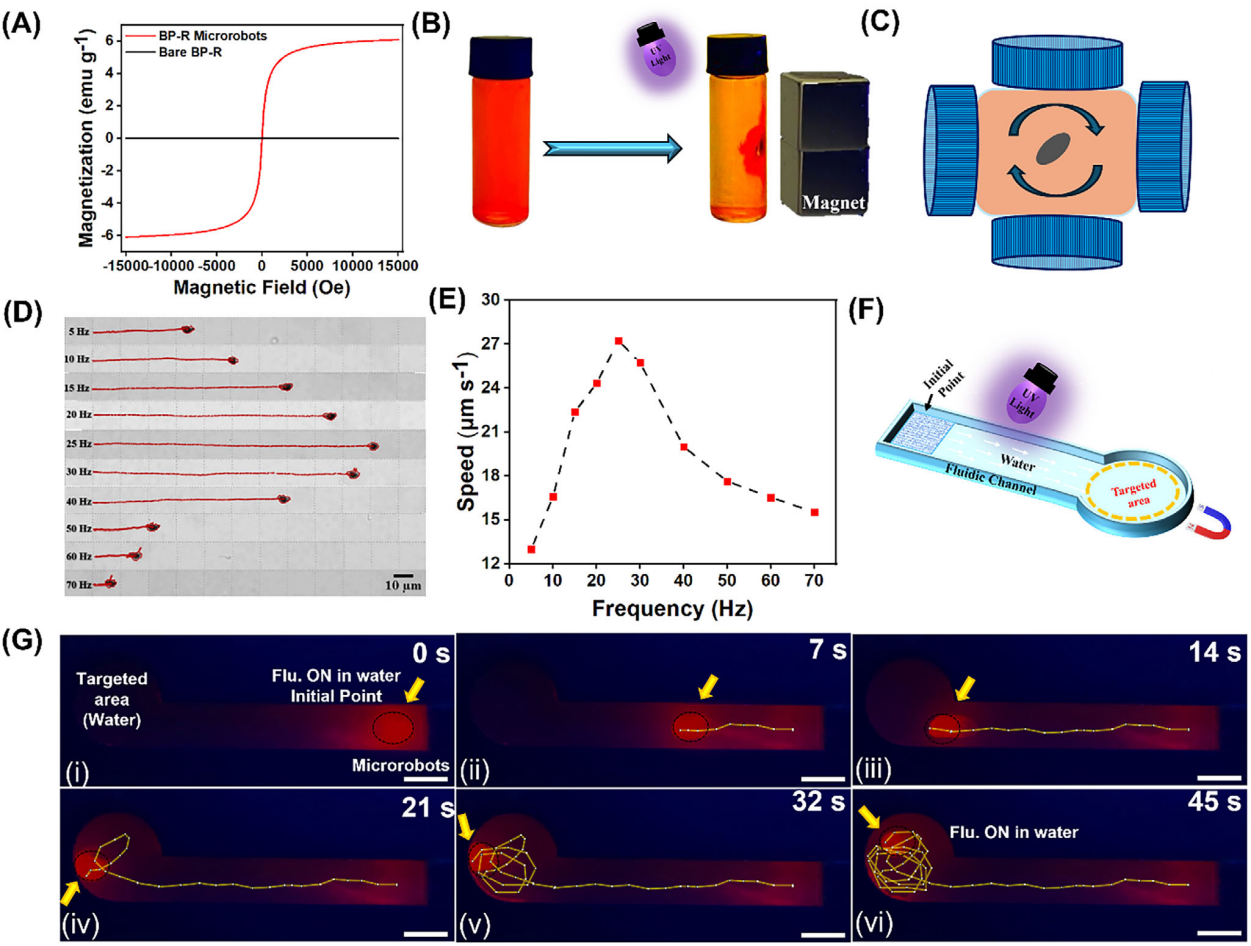
Magnetic actuation and motion of BP‐R microrobots. A) Vibrating sample magnetometer analysis of BP‐R microrobots. B) BP‐R microrobots’ magnetic collectability under UV illumination. C) Schematic illustration of the magnetic setup employed for actuation experiments. D) Displacement and E) BP‐R microrobots’ average speed measured at 5 mT across frequencies from 5 to 70 Hz (scale bar: 10 µm). F) Schematic of the fluidic channel utilized in the experiments. G) Movement of BP‐R microrobots in the fluidic channel using an external magnetic field through water.

The magnetic properties of the fabricated microrobots were characterized through magnetization measurements. The magnetic properties of BP‐R microrobots and Fe_3_O_4_ nanoparticles were studied using a vibrating sample magnetometer (VSM). The hysteresis loops, as shown in **Figure** **3**A for BP‐R microrobots and Figure S3 (Supporting Information) for Fe_3_O_4_ nanoparticles, indicate that the bare BP‐R microparticles acquired magnetic properties upon incorporation of superparamagnetic Fe_3_O_4_ nanoparticles. This functionalization allows precise control of the magnetic locomotion of the microrobots with controlled navigation. Magnetic collectability of the microrobots is illustrated in Figure 3B, which shows a neodymium–iron–boron (NdFeB) magnet being used. When the microrobots in the vial are placed near the magnet, the microrobots travel toward the magnetic field, which confirms the magnetic response of the microrobots to external stimuli. The leakage of BP‐R microrobots was evaluated by fluorescence measurements over several washing cycles. The fluorescence intensity of the microrobots remained nearly constant, while the supernatant collected after removing the microrobots showed negligible fluorescence, as shown in Figure S4 (Supporting Information), which confirms the absence of fluorescent material leakage. Stable conjugations are established between BP‐R materials and Fe_3_O_4_ nanoparticles by observing negligible leakage. The dynamic controllability of BP‐R microrobots, other than magnetic collectability, was systematically studied using a custom‐designed triaxial magnetic actuation platform developed with optical microscopy and a high‐precision control unit as shown in Figure 3C. The actuation of BP‐R microrobots in pH 7 and pH 2 was recorded by the customized magnetic setup, which consisted of three coil pairs arranged orthogonally to generate transverse rotating magnetic fields for controlling the motion of the microrobots remotely and without contact, as depicted in Movies S1 and S2 (Supporting Information), respectively. To characterize the locomotion induced by magnetic forces, microrobots were subjected to rotating magnetic fields of frequency from 5 to 70 Hz at a constant field strength of 5 mT. As shown in Figure 3D, the translational displacement of microrobots could be precisely modulated by tuning the actuation frequency. From Figure 3E, it can be determined that, at 5 mT, the average propulsion speed increased with frequency, peaking at ≈27 µm s^−1^ at the step‐out frequency of 25 Hz; thereafter, a gradual decrease in the speed is observed. As depicted in Figure S5 (Supporting Information), the average propulsion speed of microrobots has a negligible change from 27 µm ^−1^s at pH 7 to 25 µm ^−1^s at pH 2, indicating no significant impact of acidity on motion. These results confirm stable and reliable locomotion of the microrobots even in harsh acidic environments. Guided navigation was further exemplified via a microfluidic channel system (schematically shown in Figure 3F) with the actual setup depicted in Figure 3Gi). A suspension of magneto‐fluorescent microrobots, in its fluorescence “ON” state, was introduced at the inlet of the channel while the targeted reservoir was filled with deionized water. Upon the application of an external magnetic field using a handheld permanent magnet, the microrobots were guided toward the target zone, exhibiting controlled circular motion upon arrival (Figures 3Gii–vi). Throughout this navigation process, observed that the fluorescence emission remained constantly in the “ON” state under UV illumination, as confirmed by time‐lapse fluorescence images. These results reflect efficient remote‐controlled maneuverability and stable fluorescent signaling of microrobots, highlighting their potential for targeted sensing applications in complex fluidic environments, as shown in Movie S3 (Supporting Information).

### 2.3 Selective Sensing of Picric Acid by Microrobots

**Figure 4:**
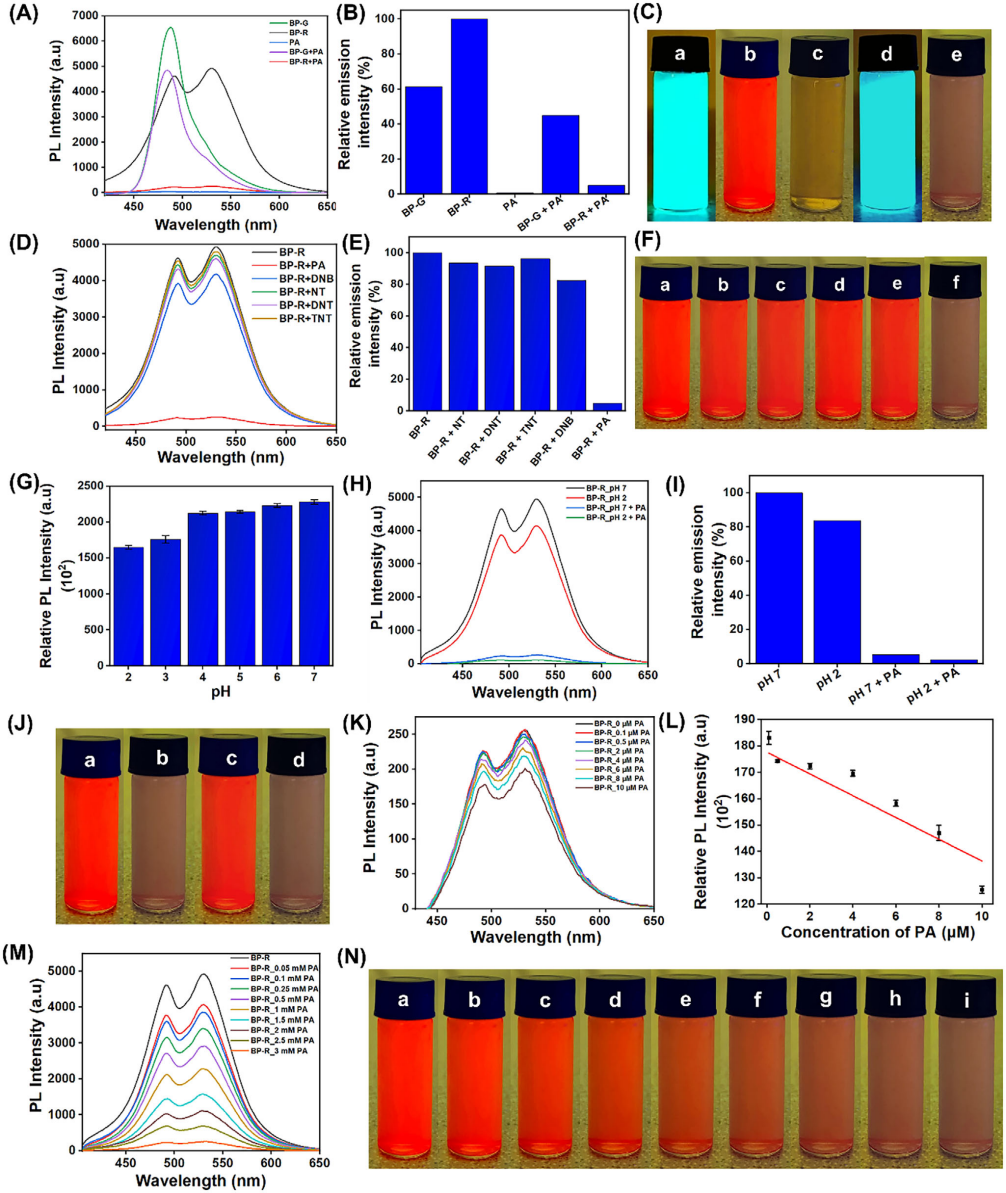
Sensing performance of microrobots. A) Photoluminescence emission spectra, B) relative emission intensity, C) images of BP‐G microparticles and BP‐R microrobots in vials, captured under UV light, pre‐ and post‐exposure to picric acid ((a) BP‐G microparticle, (b) BP‐R microrobots, (c) picric acid, d) BP‐G microparticles after picric acid treatment, e) BP‐R microrobots after picric acid treatment), (D) emission spectra, (E) relative emission intensity, F) images of BP‐R microrobots with various nitroaromatic compounds under UV illumination ((a) BP‐R microrobots, (b) BP‐R microrobots + NT, (c) BP‐R microrobots + DNT, (d) BP‐R microrobots + TNT), (e) BP‐R microrobots + DNB, (f) BP‐R microrobots + picric acid, G) relative emission intensity of BP‐R microrobots in different pH (2–7), H) emission spectra, I) relative emission intensity, J) images of BP‐R microrobots at pH 7 and pH 2 before and after picric acid treatment under UV illumination ((a) BP‐R microrobots at pH 7, (b) BP‐R microrobots at pH 7 + picric acid, (c) BP‐R microrobots at pH 2, (d) BP‐R microrobots at pH 2 + picric acid). K) Emission spectra of BP‐R molecules with increasing concentration of picric acid (0.1–10 µM) at acidic condition. L) Linear plot of PL emission intensity versus increasing concentration of picric acid. M) Emission spectra of BP‐R microrobots with different concentration of picric acid (0.1–3 mM). N) Images of BP‐R microrobots with different concentration of picric acid under UV illumination ((a) BP‐R microrobots, (b) BP‐R microrobots + 0.1 mM picric acid, (c) 0.25 mM picric acid, (d) 0.5 mM picric acid, (e) 1 mM picric acid, (f) 1.5 mM picric acid, (g) 2 mM picric acid, (h) 2.5 mM picric acid, (i) 3 mM picric acid).

The microrobots were constructed from building blocks functionalized with ketenimine groups, enabling selective interaction with target analytes for sensing applications. In this work, we use picric acid as the target molecule to evaluate the detection capability of BP‐R microrobots. The fluorescence response upon exposure to picric acid is presented in **Figures** **4**A–C. As shown in Figures 4A,B, the microrobots exhibited a significant fluorescence quenching, with an intensity loss of ≈95% after picric acid treatment. But there is very little fluorescence quenching that happens to BP‐G microparticles, confirming that the BP‐G does not have the ability to quench its fluorescence with the interaction of picric acid. As illustrated in Figure 4C, the microrobots were placed in vials and examined under UV light both before and after picric acid treatment to visually verify the results. The images clearly exhibit that the bright red fluorescence of BP‐R (Figure 4Cb) loses significantly, followed by picric acid treatment (Figure 4Ce), whereas BP‐G (Figure 4Ca) does not lose the fluorescence even after the picric acid treatment (Figure 4Cd). To assess selectivity toward picric acid, BP‐R microrobots were treated with a series of nitroaromatic explosives, such as 2,4,6‐trinitrotoluene (TNT), 1,3‐dinitrobenzene (DNB), 2,6‐dinitrotoluene (DNT), 4‐nitrotoluene (NT), and 2,4,6‐trinitrophenol (picric acid). Their photoluminescence emission spectra were systematically recorded. The photoluminescence emission spectra shown in Figures 4D,E revealed pronounced quenching only in the presence of picric acid. The high selectivity of BP‐R microrobots is due to the presence of an acidic hydroxyl group in the picric acid. That enables efficient proton transfer and effective charge‐transfer complex formation with BP‐R microrobots, leading to strong fluorescence quenching. Other nitroaromatic compounds lack the ─OH group, which prevents such interactions with BP‐R microrobots which making them highly selective toward picric acid. Molecular structures of nitroaromatic compounds used in this study are shown in Figure S6 (Supporting Information). This selective sensing capability was demonstrated by photographic images of the microrobot solutions with the various analytes, as shown in Figure 4F. To establish the selective picric acid sensing of BP‐R microrobots in acidic conditions, sensing was carried out at various acidic pH values ranging from pH 2 to 7. Figures 4G and S7 (Supporting Information) clearly show that the microrobots are compatible with monitoring picric acid in highly acidic conditions. As shown in Figures 4H,I, BP‐R microrobots exhibit strong fluorescence emissions at both pH 7 and pH 2 medium; upon the addition of picric acid, a pronounced quenching effect is observed under both conditions, where the relative PL intensity drops by ≈95% following picric acid addition at both pH conditions. These results demonstrate the strong and selective fluorescence quenching response of BP‐R microrobots toward picric acid in both neutral as well as in highly acidic conditions. For adjusting the pH, we use HCl; here, the quenching is not simply due to protonation, as the addition of HCl alone, despite its strong proton‐donating ability, does not lead to a comparable decrease in fluorescence intensity. HCl will only protonate the ketenimine moiety, but it does not have the ability to form a stable complex with it. But picric acid can protonate the ketenimine along with the formation of a charge‐transfer picrate complex, which is responsible for the observed fluorescence quenching. Thus, the specific quenching of BP‐R microrobots toward picric acid can be proven as a combination of protonation and complex formation, which is not replicated by HCl alone. Pictorial representation of the sensing picric acid in both pH conditions is shown in Figure 4J. The limit of detection (LOD) for picric acid (both acidic and neutral conditions) was calculated using the derivation: LOD = 3.3*σ/k* as shown in Figures 4K,L (acidic condition) and Figure S8 (Supporting Information) (neutral condition), where *k* is the slope of the calibration curve obtained from the plot between fluorescence intensity against picric acid with varying concentrations ranging from 0.1 to 10 µM, and *σ* is the standard deviation of fluorescence intensities determined from blank BP‐R samples (*n* = 6) prior to analyte exposure. BP‐R microrobots has a very high sensitivity, with a detection limit of 255 nm  at pH 2, indicating the trace level detection capability in acidic conditions, whereas, it shows 238 nM of detection limit in neutral conditions. The sensing performance of BP‐R microrobots in a highly acidic environment, along with other fluorescent probes, for detecting picric acid, is summarized in the comparison Table S2 (Supporting Information). The absorption spectra and photoluminescence emission spectra of BP‐R microrobots at fixed concentrations, exposed to varying concentrations of picric acid, are presented in Figures 4M and S9 (Supporting Information), respectively. The data illustrate a gradual quenching of fluorescence emission, which is paralleled by the increase in absorption, with the increase in the concentrations of picric acid, highlighting an effective sensing capability of BP‐R microrobots toward picric acid. Pictorial representation of the concentration‐dependent sensing response is depicted in Figure 4N. To evaluate the response time of BP‐R microrobots, it was treated with varying concentrations of picric acid, and their fluorescence response was recorded as shown in Figure S10 (Supporting Information). A rapid fluorescence quenching occurred within 30 s, then fluorescence intensity was stabilized, indicating the rapid sensing capability of microrobots and demonstrating the microrobots’ suitability for real‐time sensing applications. As our goal is to monitor the picric acid in real‐world scenarios, we have studied the effect of basic conditions and various common ionic species that exist in aqueous bodies on the selectivity of the microrobots. As with microrobots sensing toward picric acid in acidic conditions, their sensitivity is not changed in basic pH conditions, as shown in Figure S11 (Supporting Information). Selectivity analysis on different metal ions depicted in Figure S12 (Supporting Information) confirms that the interferents, namely, Ca^2+^, Co^2+^, Cu^2+^, Fe^2+^, K^+^, Na^+^, Ni^2+^, and Zn^2+^, which are common ionic species, did not interfere severely on account of picric acid sensing.

**Figure 5:**
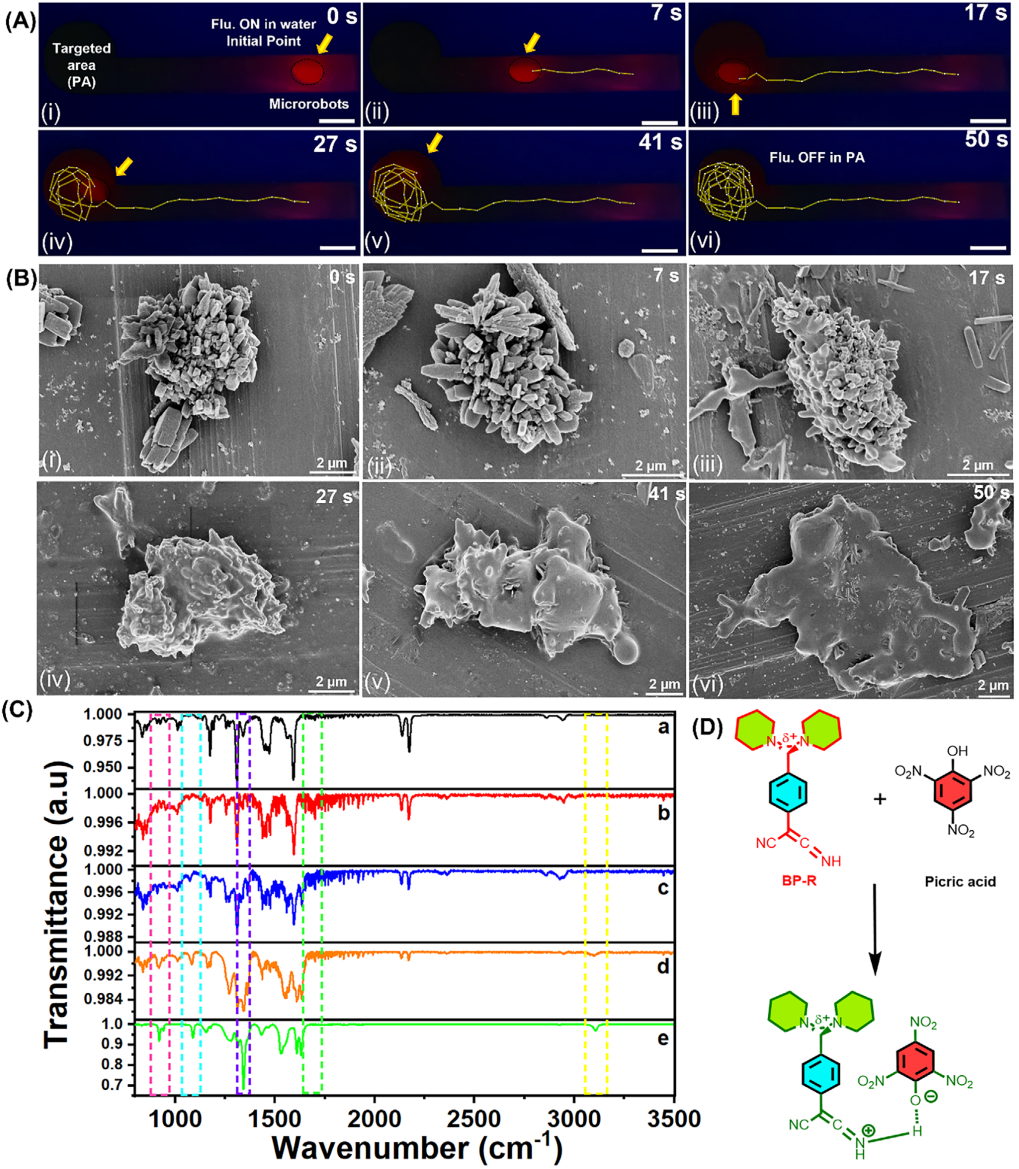
Microrobots’ sensing of explosives in a microfluidic channel. A) Movement of BP‐R microrobots in the microfluidic channel using an external magnetic field toward the target area filled with picric acid. B) Scanning electron microscopy images of microrobots in the presence of picric acid (3 µM) at different time intervals ((i) 0 s, (ii) 7 s, (iii) 17 s, (iv) 27 s, (v) 41 s, (vi) 50 s, respectively). C) Fourier‐transform infrared spectroscopy spectra of BP‐R microrobots with different concentrations of picric acid: (a) BP‐R microrobots, (b) 0.1 mM picric acid, (c) 0.5 mM picric acid, (d) 1 mM picric acid, (e) bare picric acid. D) Mechanism of microrobots’ fluorescence quenching.

For the real‐time application of developed microrobots, an artificial testing environment using a fluid channel system was developed (**Figure** **5**A). The experiments demonstrated the long‐range magnetic movement of the microrobots, which were guided several centimeters by an external magnetic force to an area containing picric acid. Time‐lapse fluorescence images under the illumination of UV light demonstrated the dynamic navigation behavior of microrobots, as shown in Movie S4 (Supporting Information). Initially, red fluorescent microrobots were placed at the starting position as shown in Figure 5Ai. They were then directed toward the picric acid‐rich target zone (Figures 5Aii–iii), performing circular trajectories once they entered the target zone (Figures 5Aiv–vi). This kind of movement would prolong contact time between microrobots and picric acid, enhancing interaction. At the end of this interaction, the fluorescence started to quench gradually until complete quenching, thus proving the effectiveness of sensing picric acid. Morphology of the microrobots was monitored continuously at different time intervals, using scanning electron microscopy to elucidate the structural change of the microrobots upon interaction with picric acid, as shown in Figures 5Bi–vi. BP‐R microrobots in water showed no morphological degeneration (Figures 5Bi,ii), thus confirming structural stability in neutral media. There was significant structural degradation of microrobots upon exposure to picric acid, and complete degradation is observed in Figures 5Biv–vi. These observations match with the earlier described fluorescence quenching and indicate a strong chemical interaction between picric acid and the microrobot matrix. Exposure to picric acid causes irreversible structural and chemical changes in BP‐R microrobots due to strong proton transfer and charge‐transfer complex formation. This permanently alters their fluorescence and morphology, as seen in the SEM images in Figure 5Bvi, since it forms a new compound with picric acid. Hence, the microrobots function as single‐use disposable sensors with a distinct on–off response. After exposing BP‐R microrobots to picric acid, the hydroxyl peak of picric acid at 3433 cm^−1^ disappeared and a new broad peak at 3090 cm^−1^ appeared, indicating proton transfer from the hydroxyl group of picric acid to the nitrogen in the ketenimine units of BP‐R through strong hydrogen‐bonding interactions, leading to the formation of charge‐transfer picrate complexes, as illustrated in Figure 5C.^[^ ^63^, ^64^, ^65^ ^]^ The peak of ketenimine in BP‐R microrobots at 1680 cm^−1^ disappeared due to the complex formation. The interaction of picric acid with BP‐R microrobot reduces the peaks of picric acid, C─O bending, and NO_2_ symmetric stretching vibrations at 1087 and 1343 cm^−1^, respectively. As discussed before, protons are transferred from picric acid to BP‐R microrobots via hydrogen bonding, leading to the formation of a charge‐transfer complex. This process redistributes the electron density within the picrate moiety, which influences the symmetric and asymmetric stretching vibrations of the NO_2_ groups in picric acid. Similarly, the C─H out‐of‐plane bending mode observed at 921 cm^−1^ in picric acid becomes reduced. Furthermore, the nitrile stretching bands of BP‐R microrobots, appearing at 2135 and 2172 cm^−1^, show no shift with increasing picric acid concentration. This indicates that the nitrile groups, although capable of electrostatic interactions, are not participating in any such interactions in this system. The fluorescence quenching mechanism is attributed to the formation of charge‐transfer complexes between the electron‐rich ketenimine groups of BP‐R microrobots and the electron‐deficient nitro groups of picric acid, as depicted in Figure 5D. This initiation occurs when the hydroxyl group of picric acid transfers a proton to the ketenimine site of BP‐R, creating water‐soluble picrate complexes, which leads to an effective fluorescence quenching.

## 3 Conclusion

In this study, we developed a rational design and successfully implemented magnetically responsive fluorescent microrobots for selective sensing of picric acid in acidic aqueous environments. The incorporation of magnetic Fe_3_O_4_ nanoparticles with ketenimine‐functionalized fluorescent small molecules makes them a multifunctional interface for molecular recognition combined with remote‐controlled navigation to confined, inaccessible, hard‐to‐access, and toxic environments. Interaction of microrobots with picric acid leads to a pronounced fluorescence quenching response due to hydrogen bonding and charge‐transfer interactions, rendering a sensitive and selective sensing of picric acid in the harsh acidic conditions. They can be dynamically maneuvered through complicated fluidic channels using an external magnetic field, which serves as a proof‐of‐concept for the real‐time scenarios and in situ sensing in hard‐to‐reach environments. The combination of switching fluorescence, molecular specificity, and magnetic actuation portrays these hybrid microrobots as intelligent mobile sensors for advanced environmental surveillance and security applications. Not only does the present work enhance the current microrobotic sensing systems, but it also marks an important step toward the development of next‐generation smart materials with respect to hazardous chemicals in a real‐time environment.

## 4 Experimental Section

### Reagents and Materials

7,7,8,8‐Tetracyanoquinodimethane (TCNQ, C_12_H_4_N_4_, 98%), piperidine (C_5_H_11_N, 99%), trifluoroacetic acid (TFA, CF_3_COOH, 99%), acetonitrile (CH_3_CN, 99.9%), sodium bicarbonate (NaHCO_3_, ≥ 99.7%), iron(II) sulfate heptahydrate (FeSO_4_∙7H_2_O, ≥ 99%), ferric chloride hexahydrate (FeCl_3_∙6H_2_O, ≥ 98%), calcium chloride dihydrate (CaCl_2_·2H_2_O, ≥ 99%), picric acid (1.3% in H_2_O, 97%), 1,3‐dinitrobenzene (DNB, 97%), 4‐nitrotoluene (NT, 99%), 2,6‐dinitrotoluene (DNT, 98%), and 2,4,6‐trinitrotoluene (TNT (10 mg mL^−1^ in acetonitrile solution), dried and dissolved in water before use) were obtained from Sigma‐Aldrich and used for experiments without further purification. Deionized (DI) water was used in the entire experiment, including the synthesis of Fe_3_O_4_ nanoparticles, preparing BP‐R microrobots, and nitroaromatic compounds.

### Synthesis of Fluorescent BP‐R Molecules

The synthesis of BP‐R molecule is shown in Figure 1A. It is a two‐step synthesis procedure. In the first step, the fluorescent core material, 7,7‐bis(piperidino)‐8,8‐dicyanoquinodimethane (BP‐G), was synthesized by adding 290 µL of piperidine to a stirred solution of TCNQ (150 mg) in 15 mL of acetonitrile at 72 °C. The mixture was stirred continuously for 3 h. Upon addition of piperidine, the initially brownish‐yellow solution of TCNQ eventually turns green. The resulting green precipitate was filtered, thoroughly washed with deionised water, and dried under vacuum, and the resulting crystals were used for single‐crystal X‐ray diffraction (SCXRD) analysis.

In the second step, BP‐G (4 mg) was treated with trifluoroacetic acid (TFA) through the stepwise addition of 100 µL for 4 times at 5‐min intervals over a total period of 20 min while maintaining the reaction temperature at 130 °C. Upon each addition of TFA, the BP‐G gradually transforms, ultimately turning deep red, indicating the formation of the ketenimine‐based product, BP‐R. The obtained BP‐R was further dissolved in 1 m hydrochloric acid and subsequently recrystallized using a saturated solution of sodium bicarbonate. The recrystallized BP‐R was then utilized for the fabrication of microrobots.

### Preparation of Magnetically Responsive Fe_3_O_4_ Nanoparticles

Magnetite (Fe_3_O_4_) nanoparticles have been synthesized by the co‐precipitation method under mild aqueous conditions. Briefly, an aqueous solution of 20 mg of calcium chloride dihydrate (CaCl_2_∙2H_2_O) and 38 mg of ferrous sulfate heptahydrate (FeSO_4_∙7H_2_O) was prepared in 20 mL of deionized water each. Both solutions were mixed for 5 min at room temperature (25 °C) under constant stirring to ensure homogeneous mixing. Afterward, 37 mg of ferric chloride hexahydrate (FeCl_3_∙6H_2_O) was added to the mixture, heated at 60 °C, and stirred for an additional 15 min to allow precursor interaction. Ammonium hydroxide (NH_4_OH) (30%) solution was added dropwise to raise the pH of the reaction medium to ≈11, and the mixture was stirred vigorously for another 15 min at 60 °C to ensure complete precipitation of Fe_3_O_4_ nanoparticles. The black precipitate thus formed was separated from the solution using a permanent magnet and then washed with deionized water and ethanol to eliminate unreacted salts and other residual impurities. The washed Fe_3_O_4_ nanoparticles were then dried overnight under ambient conditions and stored under vacuum for further use. The magnetic nanoparticles were selected in this instance for microrobotic architectures due to their excellent magnetic responsiveness and aqueous dispersibility.

### Assembly of BP‐R/Fe_3_O_4_‐Based Microrobots

As described in Section 4.2, the obtained BP‐R compound (4 mg) was further dissolved in 1 m hydrochloric acid (500 µL), followed by 1.5 mL of aqueous solution of Fe_3_O_4_ nanoparticles (1 mg in 10 mL of water) was added and left undisturbed for 30 min. Then, the solution was treated with sodium bicarbonate to regenerate the micro‐sized crystals while Fe_3_O_4_ NPs were decorated on the surface of BP‐R microcrystals. The resulting Fe_3_O_4_ NP‐decorated BP‐R microcrystals were collected by using a permanent magnet and then employed for actuation studies and sensing applications. Pictorial representation of the synthesis of microrobots is shown in Figure S2 (Supporting Information).

### Optical and Spectroscopic Characterization

Electronic absorption and photoluminescence emission spectra were measured using a Jasco V‐750 UV–Visible spectrophotometer and Jasco FP‐8300 spectrofluorometer, respectively. Spectral data were recorded for the solution and microcrystalline forms of BP‐G, BP‐R, and BP‐R microrobots. Fluorescence measurements were measured continuously at an excitation wavelength of 350 nm. For solution‐phase studies, acetonitrile solutions of BP‐G and BP‐R (0.1 mM) were used to record their respective absorption spectra and photoluminescence emission spectra. The solid‐state fluorescence and absorption characteristics were evaluated using microcrystalline samples of BP‐G and BP‐R. For the sensing performance studies on BP‐R microrobots with picric acid, the fluorescence measurements were employed. The experiment involved mixing 0.5 mL of BP‐R microrobot dispersion with 0.5 mL of aqueous picric acid solutions at different increasing concentrations (0.05, 0.1, 0.25, 0.5, 1, 1.5, 2, 2.5, and 3 mM) and diluting to 3 mL with deionised water. The resulting emission spectra were recorded to monitor the emission quenching behavior. To evaluate selectivity, BP‐R microrobots (0.5 mL) were mixed with 0.5 mL of aqueous solutions (3 mM) of various nitroaromatic compounds, including 2,4,6‐trinitrotoluene (TNT), 1,3‐dinitrobenzene (DNB), 2,6‐dinitrotoluene (DNT), 4‐nitrotoluene (NT), and picric acid, and diluted to 3 mL with deionised water. The emission spectra were compared to establish analyte‐specific quenching profiles. Studies were done to understand the role of environmental pH on the sensing behavior of microrobots, involving the mixing of 0.5 mL of BP‐R microrobots and 0.5 mL of 1 mM picric acid, followed by mixing with 2 mL of varying pH aqueous solutions ranging from 2 to 12 (achieved using HCl or NaOH). For the sensing performance of microrobots in highly acidic (pH 2) conditions, a mixture of 0.5 mL of BP‐R microrobots and 0.5 mL of 3 mM picric acid was diluted to 3 mL with pH 2 aqueous solutions and recorded the emission spectra. The response time of BP‐R microrobots was evaluated by monitoring their interaction with varying concentrations of picric acid (0.1, 0.5, 1, 2, and 3 mM) over a period ranging from 30 s to 10 min. The influence of different ionic species on fluorescence performance was also evaluated by preparing mixtures containing 0.5 mL of BP‐R microrobots, 0.5 mL of 1 mM picric acid, and 0.5 mL of 1 mM aqueous solutions of various ionic species (Na^+^, K^+^, Mg^2+^, Ca^2+^, Cl^−^, SO_4_^2+^) and diluted with 2 mL of deionised water, after which the emission spectra were recorded for each sample. For limit‐of‐detection (LOD) analysis, the stock dispersion of the microrobot was made as described in Section 4.4. An aliquot (10 µL) of this stock was added to 3 mL of aqueous picric acid solutions with increasing concentrations ranging from 0.1 to 10 µM (0.1, 0.5, 2, 4, 6, 8, and 10 µM). FTIR spectroscopy was performed using a Bruker Vertex 70v spectrometer coupled with a Hyperion 3000 IR microscope. FTIR analysis was carried out by treating 0.5 mL of picric acid solutions with 0.5 mL of BP‐R microrobots at various concentrations to find the characteristic spectral shifts in vibrations of functional groups, which confirmed the interaction of picric acid with the sensing matrix.

### Magnetic Actuation and Locomotion Analysis

The custom‐made magnetic actuation system, having three pairs of electromagnetic coils aligned orthogonally, was mounted on a poly (lactic acid) (PLA)‐based 3D‐printed frame for actuation assessment of the microrobots. The system is interfaced with a programmable navigation controller, which means it can finely control the modulation of field strength and frequency of actuation. The experiments were performed under a homogeneous magnetic field of 5 mT with excitation frequencies varying between 5 and 70 Hz. The locomotion of microrobots at pH 7 and pH 2 was recorded employing a Nikon Ts2R inverted optical microscope positioned with a Basler acA1920‐155uc high‐speed camera while recording videos at 20 frames/s, as shown in Movie S1 and Movie S2 (Supporting Information), respectively. All the motion experiments were performed in aqueous media containing sodium dodecyl sulfate (SDS, 0.25 wt.%) to prevent aggregation and maintain good dispersion. The analysis of the trajectories and velocities of the microrobots was performed using NIS‐Elements Advanced Research Software and FIJI (ImageJ). By tracking displacements of multiple microrobots over time with different actuation frequencies, the average translational velocity was calculated. Therefore, frequency‐dependent propulsion characteristics become important in formulating the concept of dynamic response to optimize the microrobots’ performance in sensing and navigation applications.

### Structural Characterization via X‐Ray Diffraction

SCXRD analysis of BP‐G was performed by a Rigaku Oxford XtaLAB Synergy‐R diffractometer, which has a hybrid pixel array detector and automatic crystal transport, orientation, and retrieval (ACTOR) system. Cu Kα radiation generated from the rotating anode X‐ray source was used to collect data at 120 K. Raw diffraction frames were processed with CrysAlisPro v1.171.42.95a, and SHELXL‐2019/3 was used for structure solution and refinement, respectively. The generated crystallographic information was employed in establishing the molecular structure of BP‐G with a high degree of precision. PXRD profiles of BP‐G, BP‐R microcrystals, and BP‐R microrobots were recorded using the Rigaku SmartLab 3 kW diffractometer working at 40 kV and 30 mA. Experimental PXRD profiles were compared with the simulated diffraction patterns generated from SCXRD data using Mercury 3.8 software to assess the crystallinity and phase purity of all samples. Integration of SCXRD and PXRD analyses allowed detailed structure validation of the synthesized materials and confirmed the structural and crystalline nature of the material.

### Morphological and Elemental Analysis by Electron Microscopy

Morphological characterization and element composition of BP‐R microrobots were done through field emission scanning electron microscopy (FESEM) and energy‐dispersive X‐ray spectroscopy (EDX). High‐resolution surface imaging was done using FEI Verios 460L FESEM. The elemental mapping as well as composition analyses were done using a Tescan MIRA3 XMU SEM. For analysis, microrobots were drop‐cast on a conductive copper tape and coated with a 10 nm gold layer utilizing a high vacuum sputter coater from Leica EM ACE600 to enhance electron conductivity and image resolution. Fluorescence microscopy was used to appraise the optical properties of microrobots. Imaging was done using a Nikon Ts2R inverted fluorescence microscope with a CoolLED pE‐100 UV LED source, along with a Basler acA1920‐155uc digital camera. The DAPI emission filter and 40× and 60× objectives were used to obtain high‐resolution fluorescence micrographs. All analyses show that BP‐R microrobots do have structural uniformity and fluorescent response, thus validating their actuation and sensing.

### Real‐Time Targeted Sensing in Fluidic Microenvironments

For demonstrating the dynamic‐sensing capability of BP‐R microrobots under confined and controlled fluidic conditions, a Teflon‐based microfluidic channel was chosen as the model platform. Herein, the use of a fluidic channel functions as a proof‐of‐concept platform for demonstrating the remote navigation and selective detection capabilities of BP‐R microrobots in a real‐time scenario. This channel was initially filled with 5 mL of deionized water to maintain a stagnant aqueous environment (Movie S3, Supporting Information). 1 mL of BP‐R microrobots was injected from the inlet region of the channel while a different spatially resolved target site was introduced with 1 mL of an aqueous solution of 3 mM picric acid. Actuation and directed propulsion of the microrobots toward the target area were achieved by an external permanent magnet. Upon arrival at the target area, real‐time fluorescence changes were monitored under UV illumination (365 nm) to visualize the quenching of fluorescence (Movie S4, Supporting Information). The modulation of fluorescence due to the interaction between BP‐R microrobots and picric acid allowed the selective detection of target molecules to be visualized under working conditions. The actuation of BP‐R microrobots relies on Fe_3_O_4_ nanoparticles for magnetic responsiveness and an external rotating magnetic field for propulsion. Without either of these components, the particles show no controlled locomotion. These experiments validate the capability of BP‐R microrobots for spatially resolved, magnetically guided chemical sensing in fluidic microenvironments.

## Conflict of Interest

The authors declare no competing interests.

## Author Contributions

M.P. conceptualized the project. N.T.M. and N.S. conceptualized and designed the project methodology. N.T.M. synthesized and characterized the microrobots, analyzed the data, and prepared the original manuscript draft. N.S. conducted XRD and EDAX analyses, contributed to data interpretation, revised the manuscript, and provided mentorship. M.P. supervised the project and provided overall direction. All authors contributed to the experimental design and manuscript preparation.

## Supporting information



Supporting Information

Supplemental Movie 1

Supplemental Movie 2

Supplemental Movie 3

Supplemental Movie 4

## Data Availability

The data that support the findings of this study are available in the supplementary material of this article.

## References

[1] J. Wang , Electroanalysis 2007, 19, 415.

[2] K. C. To , S. Ben‐Jaber , I. P. Parkin , ACS Nano 2020, 14, 10804. 32790331 10.1021/acsnano.0c01579

[3] R. Liu , Z. Li , Z. Huang , K. Li , Y. Lv , TrAC Trends Anal. Chem. 2019, 118, 123.

[4] J. Wang , M. Pumera , M. P. Chatrathi , A. Escarpa , M. Musameh , G. Collins , A. Mulchandani , Y. Lin , K. Olsen , Anal. Chem. 2002, 74, 1187. 11924983

[5] J. H. Song , D. W. Kang , Coord. Chem. Rev. 2023, 492, 215279.

[6] S. Giannoukos , B. Brkić , S. Taylor , A. Marshall , G. F. Verbeck , Chem. Rev 2016, 116, 8146. 27388215 10.1021/acs.chemrev.6b00065

[7] S. C. P. A. I.n Gad, Encyclopedia of Toxicology, 3rd ed., Ed. P. Wexler , Academic Press, Cambridge, Massachusetts 2014, Vol. 3, p. 952–954.

[8] I. Khan , T. Shah , M. R. Tariq , M. Ahmad , B. Zhang , J. Environ. Chem. Eng 2024, 12, 112720.

[9] Neha , N. Kaur , Environmental Surfaces and Interfaces 2024, 2, 49.

[10] B. Pramanik , S. Das , D. Das , Chem. Asian J. 2020, 15, 4291. 33137228 10.1002/asia.202001184

[11] J. F. Wyman , M. P. Serve , D. W. Hobson , L. H. Lee , D. E. Uddin , J. Toxicol. Environ. Health 1992, 37, 313. 1404487 10.1080/15287399209531672

[12] P. Kovacic , R. Somanathan , J. Appl. Toxicol 2014, 34, 810. 24532466 10.1002/jat.2980

[13] K. S. Ju , R. E. N. C. Parales , S. Biodegradation , Microbiol Mol Biol Rev 2010, 74, 250. 20508249 10.1128/MMBR.00006-10 PMC2884413

[14] M. Pumera , Electrophoresis 2008, 29, 269. 18058771 10.1002/elps.200700394

[15] B. Wang , X. L. Lv , D. Feng , L. H. Xie , J. Zhang , M. Li , Y. Xie , J. R. Li , H. C. Zhou , J. Am. Chem. Soc. 2016, 138, 6204. 27090616 10.1021/jacs.6b01663

[16] S. G. Liu , D. Luo , N. Li , W. Zhang , J. L. Lei , N. B. Li , H. Q. Luo , ACS Appl. Mater. Interfaces. 2016, 8, 21700. 27471907 10.1021/acsami.6b07407

[17] H. Cavaye , A. R. G. Smith , M. James , A. Nelson , P. L. Burn , I. R. Gentle , S. C. Lo , P. Meredith , Langmuir 2009, 25, 12800. 19610640 10.1021/la9017689

[18] L. Lin , M. Rong , S. Lu , X. Song , Y. Zhong , J. Yan , Y. Wang , X. Chen , Nanoscale 2015, 7, 1872. 25522688 10.1039/c4nr06365a

[19] Z. M. S. H. Khan , S. Saifi , Shumaila , Z. Aslam , S. A. Khan , M. Zulfequar , J. Photochem. Photobiol. A: Chem. 2020, 388, 112201.

[20] Y. Li , L. Feng , W. Yan , I. Hussain , L. Su , B. Tan , Nanoscale 2019, 11, 1286. 30603761 10.1039/c8nr07142j

[21] R. Ahmed , F. Ali , Int. Multidiscip. Res. J. 2023, 06, 1119.

[22] A. Chowdhury , P. S. Mukherjee , J. Org. Chem. 2015, 80, 4064. 25822377 10.1021/acs.joc.5b00348

[23] M. Fabin , M. Łapkowski , T. Jarosz , Appl. Sci 2023, 13, 3991.

[24] M. Urso , M. Ussia , M. Pumera , Nat. Rev. Bioeng 2023, 1, 236. 37064655 10.1038/s44222-023-00025-9 PMC9901418

[25] X. Ju , C. Chen , C. M. Oral , S. Sevim , R. Golestanian , M. Sun , N. Bouzari , X. Lin , M. Urso , J. S. Nam , Y. Cho , X. Peng , F. C. Landers , S. Yang , A. Adibi , N. Taz , R. Wittkowski , D. Ahmed , W. Wang , V. Magdanz , M. Medina‐Sánchez , M. Guix , N. Bari , B. Behkam , R. Kapral , Y. Huang , J. Tang , B. Wang , K. Morozov , A. Leshansky , et al., ACS Nano 2025, 19, 24174. 40577644 10.1021/acsnano.5c03911 PMC12269370

[26] C. M. Oral , M. Pumera , Nanoscale 2023, 15, 8491. 37186253 10.1039/d3nr00502j

[27] B. Zhu , A. Salehi , L. Xu , W. Yuan , T. Yu , Adv. Intell. Syst. 2025, 7, 2400779.

[28] B. Jurado‐Sánchez , J. Wang , Environ. Sci. Nano 2018, 5, 1530.

[29] J. Parmar , D. Vilela , K. Villa , J. Wang , S. Sánchez , J. Am. Chem. Soc. 2018, 140, 9317. 29969903 10.1021/jacs.8b05762

[30] H. Hussein , A. Damdam , L. Ren , Y. Obeid Charrouf , J. Challita , M. Zwain , H. Fariborzi , Adv. Intell. Syst. 2023, 5, 2300168.

[31] G. Gardi , S. Ceron , W. Wang , K. Petersen , M. Sitti , Nat. Commun. 2022, 13, 2239. 35473915 10.1038/s41467-022-29882-5 PMC9043221

[32] X. Peng , M. Urso , M. Ussia , M. Pumera , ACS Nano 2022, 16, 7615. 35451832 10.1021/acsnano.1c11136

[33] S. G. Ullattil , M. Pumera , Small 2023, 19, 2301467. 10.1002/smll.202301467 37309271

[34] H. Eskandarloo , A. Kierulf , A. Abbaspourrad , Nanoscale 2017, 9, 12218. 28809422 10.1039/c7nr05166b

[35] Y. Ying , A. M. Pourrahimi , C. L. Manzanares‐Palenzuela , F. Novotny , Z. Sofer , M. Pumera , Small 2020, 16, 1902944. 10.1002/smll.201902944 31464380

[36] H. Zhou , C. C. Mayorga‐Martinez , S. Pané , L. Zhang , M. Pumera , Chem. Rev 2021, 121, 4999. 33787235 10.1021/acs.chemrev.0c01234 PMC8154323

[37] P. Mayorga‐Burrezo , C. C. Mayorga‐Martinez , M. Kuchař , M. Pumera , Small 2024, 20, 2306943. 10.1002/smll.202306943 38239086

[38] H. Liu , Q. Guo , W. Wang , T. Yu , Z. Yuan , Z. Ge , W. Yang , Rev. Adv. Mater. Sci. 2023, 62, 20230119.

[39] J. G. S. Moo , C. C. Mayorga‐Martinez , H. Wang , B. Khezri , W. Z. Teo , M. Pumera , Adv. Funct. Mater. 2017, 27, 1604759.

[40] K. Han , A. Snezhko , ACS Nano 2025, 19, 16248. 40292636 10.1021/acsnano.5c01238

[41] M. T. Máthé , H. Nishikawa , F. Araoka , A. Jákli , P. Salamon , Nat. Commun. 2024, 15, 6928. 39164266 10.1038/s41467-024-50226-y PMC11336208

[42] X. Lu , K. Zhao , W. Liu , D. Yang , H. Shen , H. Peng , X. Guo , J. Li , J. Wang , ACS Nano 2019, 13, 11443. 31425653 10.1021/acsnano.9b04930

[43] Y. Zhou , H. Wang , Z. Ma , J. K. W. Yang , Y. Ai , Adv. Mater. Technol. 2020, 5, 2000323.

[44] C. Cao , F. Mou , M. Yang , S. Zhang , D. Zhang , L. Li , T. Lan , D. Xiao , W. Luo , H. Ma , J. Guan , Adv. Sci. 2024, 11, 2401711. 10.1002/advs.202401711 PMC11321641 38868929

[45] M. Urso , M. Ussia , X. Peng , C. M. Oral , M. Pumera , Nat. Commun. 2023, 14, 6969. 37914692 10.1038/s41467-023-42674-9 PMC10620202

[46] L. Zhang , S. Wang , Y. Hou , ACS Nano 2025, 19, 7444. 39970007 10.1021/acsnano.4c10382

[47] S. Dutta , S. Noh , R. S. Gual , X. Chen , S. Pané , B. J. Nelson , H. Choi , Nano‐Micro Lett. 2024, 16, 41. 10.1007/s40820-023-01259-3 PMC10689718 38032424

[48] M. Ussia , M. Urso , M. Kratochvilova , J. Navratil , J. Balvan , C. C. Mayorga‐Martinez , J. Vyskocil , M. Masarik , M. Pumera , Small 2023, 19, 2208259. 10.1002/smll.202208259 36703532

[49] X. Peng , C. M. Oral , M. Urso , M. Ussia , M. Pumera , ACS Appl. Mater. Interfaces. 2025, 17, 3608. 39745814 10.1021/acsami.4c18360 PMC11744513

[50] M. Ussia , M. Urso , C. M. Oral , X. Peng , M. Pumera , ACS Nano 2024, 18, 13171. 38717036 10.1021/acsnano.4c02115 PMC11112980

[51] C. M. Oral , M. Ussia , M. Pumera , J. Phys. Chem. C. 2021, 125, 18040.

[52] X. Peng , M. Urso , M. Pumera , npj Clean Water 2023, 6, 21.

[53] X. Peng , M. Urso , M. Pumera , Small Methods 2021, 5, 2100617. 10.1002/smtd.202100617 34927942

[54] A. M. Pourrahimi , K. Villa , Y. Ying , Z. Sofer , M. Pumera , ACS Appl. Mater. Interfaces. 2018, 10, 42688. 30500156 10.1021/acsami.8b16217

[55] F. Novotný , J. Plutnar , M. Pumera , Adv. Funct. Mater. 2019, 29, 1903041.

[56] Y. Ma , Y. Zhang , X. Liu , Q. Zhang , L. Kong , Y. Tian , G. Li , X. Zhang , J. Yang , Dyes Pigments 2019, 163, 1.

[57] A. Patra , T. P. Radhakrishnan , Chem. ‐ Eur. J. 2009, 15, 2792. 19072965 10.1002/chem.200801878

[58] N. Mahajan , T. P. Radhakrishnan , J. Mater. Chem. C 2024, 12, 13430.

[59] N. Senthilnathan , K. Gaurav , C. Venkata Ramana , T. P. Radhakrishnan , J. Mater. Chem. B 2020, 8, 4601. 32391841 10.1039/d0tb00470g

[60] N. Senthilnathan , C. M. Oral , A. Novobilsky , M. Pumera , Adv. Funct. Mater. 2024, 34, 2401463.

[61] N. Senthilnathan , C. M. Oral , M. Pumera , ACS Appl. Mater. Interfaces. 2025, 17, 21691. 40145509 10.1021/acsami.5c02259 PMC11986900

[62] S. Jayanty , T. P. Radhakrishnan , Chem. ‐ Eur. J. 2004, 10, 791. 14767945 10.1002/chem.200305123

[63] S. Jayanty , T. P. Radhakrishnan , Chem. Mater 2001, 13, 2072.

[64] A. S. Gaballa , A. S. Amin , Spectrochim. Acta ‐ A: Mol. Biomol. Spectrosc. 2015, 145, 302. 25795603 10.1016/j.saa.2015.03.005

[65] T. Dhanabal , G. Amirthaganesan , M. Dhandapani , S. K. Das , J Chem Sci 2012, 124, 951.

