# Supplementary material for: Molecularly Engineered Fluorescent Magnetic Microrobots for Sensing High‐Energy Nitroaromatic Explosives in Highly Acidic Aqueous Environments
Source: Small. 2025 Dec 9;22(5):e12670. doi: 10.1002/smll.202512670 (PMC12824556; doi:10.1002/smll.202512670)
Supplement: Supplementary file 1 — Supporting Information [file SMLL-22-e12670-s005.docx]

**Supplementary Information**

**Molecularly Engineered Fluorescent Magnetic Microrobots for Sensing High-energy Nitroaromatic Explosives in Highly Acidic Aqueous Environments**

Nikhil Thekkedath Madhu,^1^ N. Senthilnathan,^1^ Martin Pumera*^1,2,3,4^

^1^Future Energy and Innovation Laboratory, Central European Institute of Technology, Brno University of Technology, Purkynova 123, Brno 61200, Czech Republic

^2^Advanced Nanorobots & Multiscale Robotics Laboratory, Faculty of Electrical Engineering and Computer Science, VSB - Technical University of Ostrava, 17. listopadu 2172/15, 70800 Ostrava, Czech Republic

^3^Department of Medical Research, China Medical University Hospital, China Medical University, No. 91 Hsueh-Shih Road, Taichung, Taiwan

^4^Department of Chemical and Biomolecular Engineering, Yonsei University, 50 Yonsei-ro, Seodaemun-gu, Seoul 03722, Korea

*✉ email:* [*martin.pumera@ceitec.vutbr.cz*](mailto:martin.pumera@ceitec.vutbr.cz)

| **Page** | **Content** |
| --- | --- |
| S3 | Nuclear magnetic resonance spectroscopic characterization of BP-G **(Figure S1).** |
| S4 | Basic crystallographic data of BP-G **(Table S1)**. |
| S5 | Preparation of BP-R microrobots **(Figure S2).** |
| S6 | Vibrating sample magnetometer analysis of Fe_3_O_4_ nanoparticles **(Figure S3).** |
| S7 | Leakage of BP-R Microrobots **(Figure S4).** |
| S8 | Speed of BP-R microrobots in acidic conditions (pH 2) **(Figure S5).** |
| S9 | Molecular structure of nitro compounds used in this study **(Figure S6)**. |
| S10 | Emission spectra of BP-R microrobots in acidic pH (2-7) **(Figure S7).** |
| S11 | Limit of detection for Picric acid at pH 7 **(Figure S8).** |
| S12 | Electronic absorption spectra of BP-R microrobots in picric acid **(Figure S9).** |
| S13 | Response time of microrobots **(Figure S10).** |
| S14 | Effect of basic pH condition on the sensing of BP-R microrobots to picric acid **(Figure S11).** |
| S15 | Effect of metal ions on the sensing of BP-R microrobots to picric acid **(Figure S12).** |
| S16 | Comparison of the sensing performance of BP-R microrobots with other fluorescent sensing probes to picric acid **(Table S2)**. |
| S17 | References. |

**Nuclear Magnetic Resonance**

**
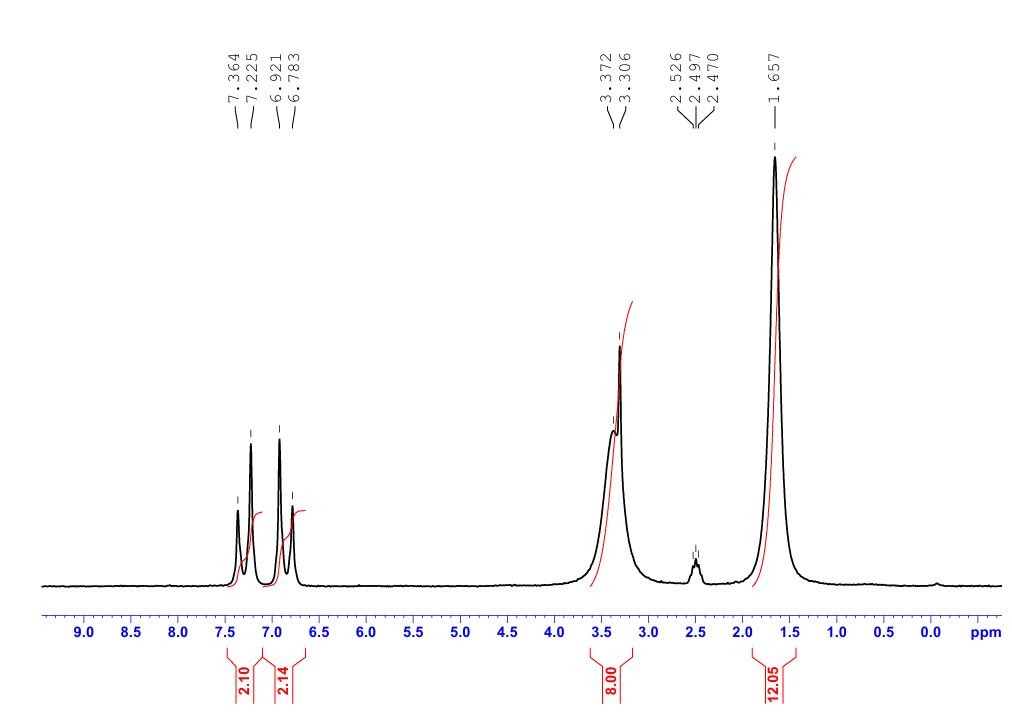
**

**Figure S1.** Nuclear magnetic resonance spectra of BP-G.

**Crystallographic data of BP-G**

|  | **BP-G** |
| --- | --- |
| Empirical formula | C_20_H_24_N_4_ |
| Crystal system | triclinic |
| Space group | *P-1* |
| a / Å | 8.70485(6) |
| b / Å | 13.40539(8) |
| c / Å | 16.13893(10) |
| α / deg. | 95.5128(5) |
| β / deg. | 101.9996(6) |
| γ / deg. | 104.5247(6) |
| V / Å^3^ | 1761.28(2) |
| Z | 4 |
| ρ_calc._ / g cm^-3^ | 1.208 |
| μ / mm^-1^ | 0.569 |
| Temperature / K | 120.01 (10) |
| λ / Å | 1.54184 |
| No. of reflections | 7274 |
| No. of parameters | 866 |
| Max., Min. transmission | 0.927,1.00 |
| GOF | 1.2822 |
| R [for I ≥ 2σ_I_] | 0.0107 |
| wR^2^ | 0.0266 |
| Largest difference peak and hole / eÅ^-3^ | 0.212/ -0.263 |
| CCDC number | 2480624 |

**Table S1**. Basic crystallographic data of BP-G.

**Preparation of BP-R microrobots**

**
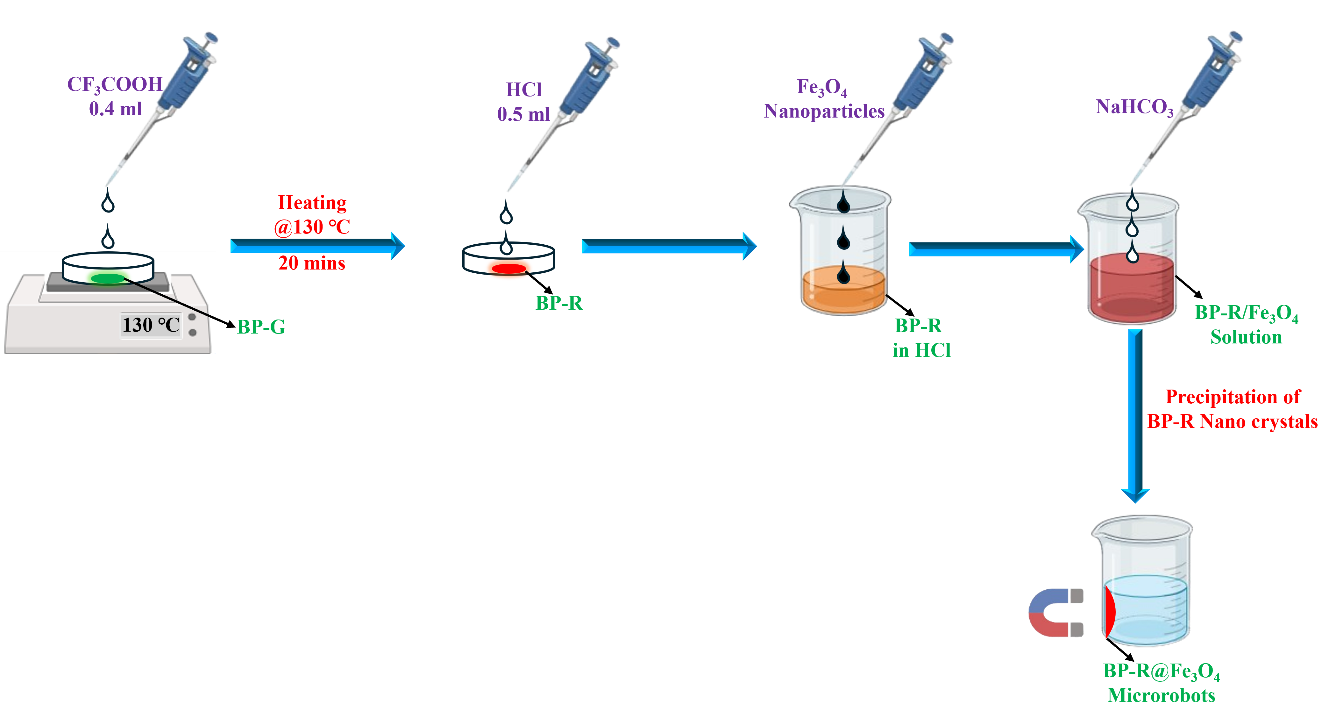
**

**Figure S2.** A pictorial representation of the preparation of BP-R microrobots.

**Vibrating Sample Magnetometer Analysis**

**
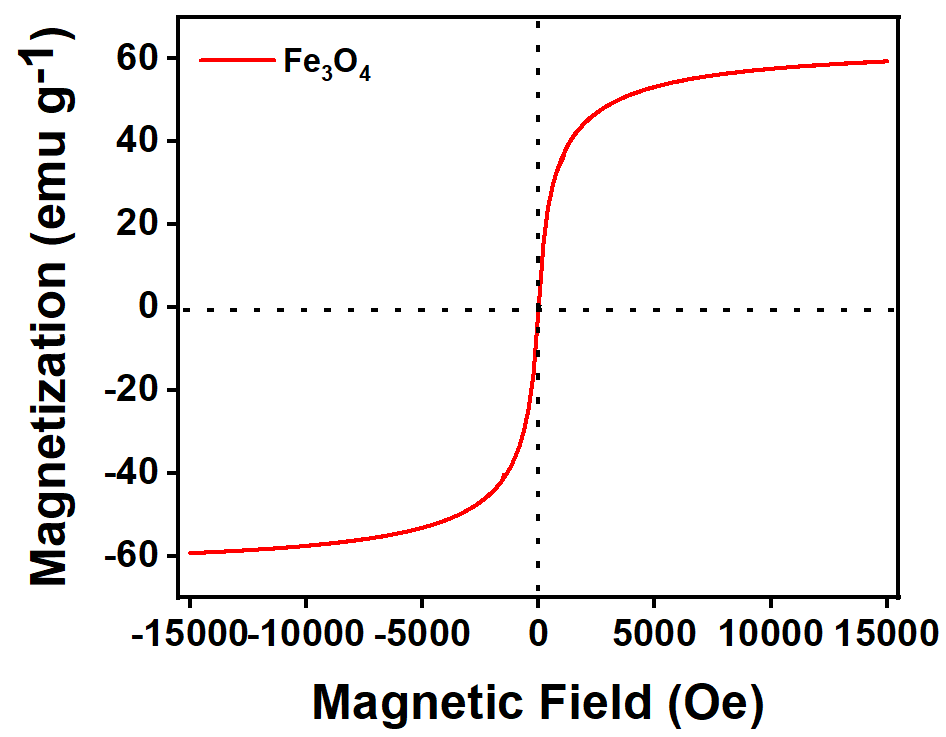
**

**Figure S3.** Vibrating sample magnetometer analysis of Fe_3_O_4_ nanoparticles used for the fabrication of microrobots in this study.

**Leakage of BP-R Microrobots**

**
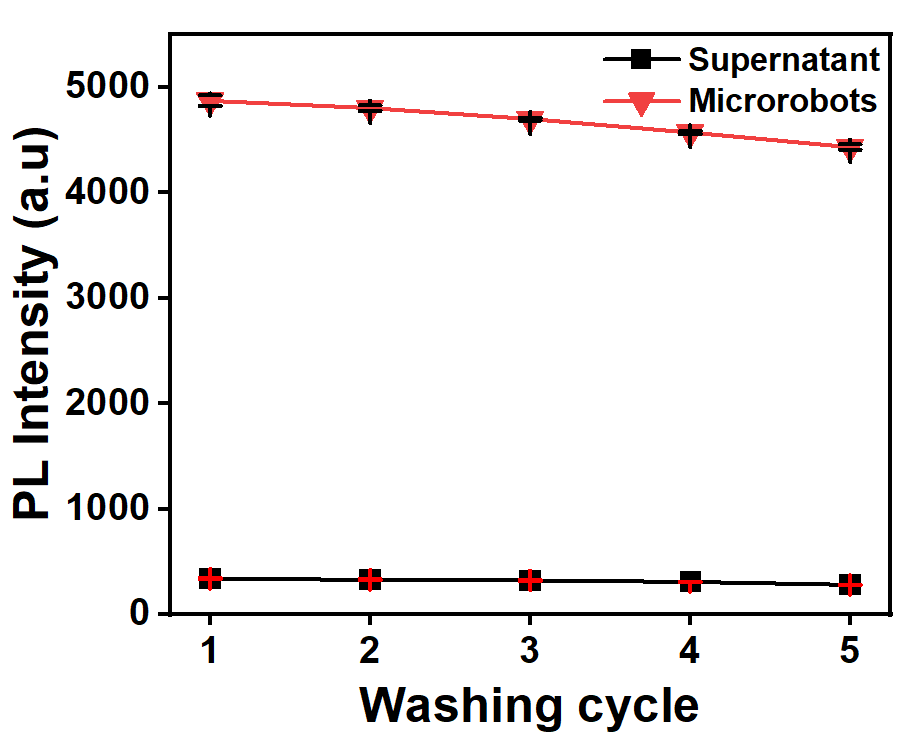
**

**Figure S4.** Fluorescence intensity of BP-R microrobots and their supernatant over five consecutive washing cycles.

**Speed of BP-R microrobots in acidic conditions (pH 2)**

**
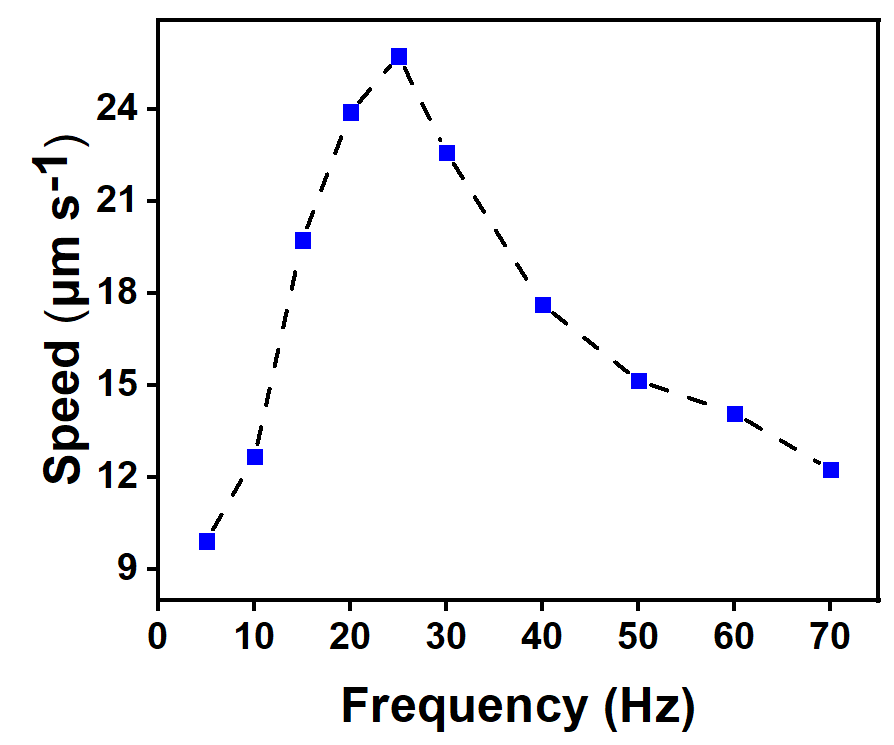
**

**Figure S5.** BP-R microrobots’ average speed measured at 5 mT across frequencies from 5 to 70 Hz in acidic aqueous solution (pH 2).

**Molecular structures of nitroaromatic explosives**

**
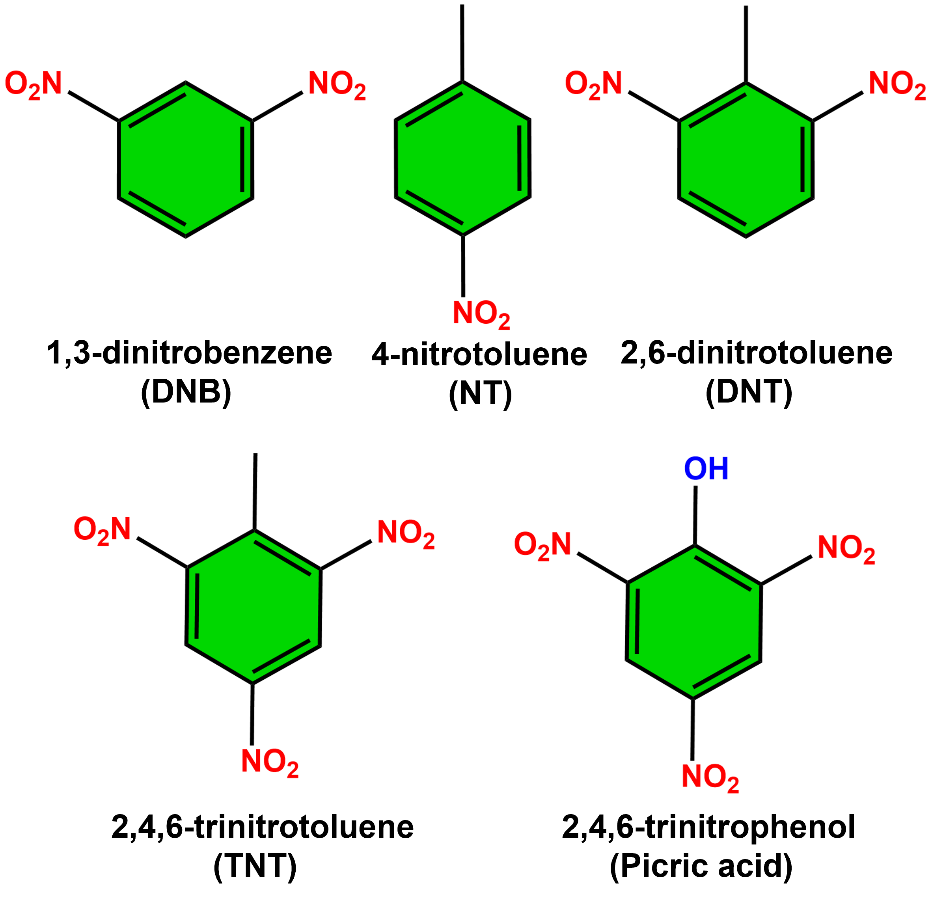
**

**Figure S6.** Molecular structure of nitro compounds used in this study.

**Fluorescence emission spectra**

**
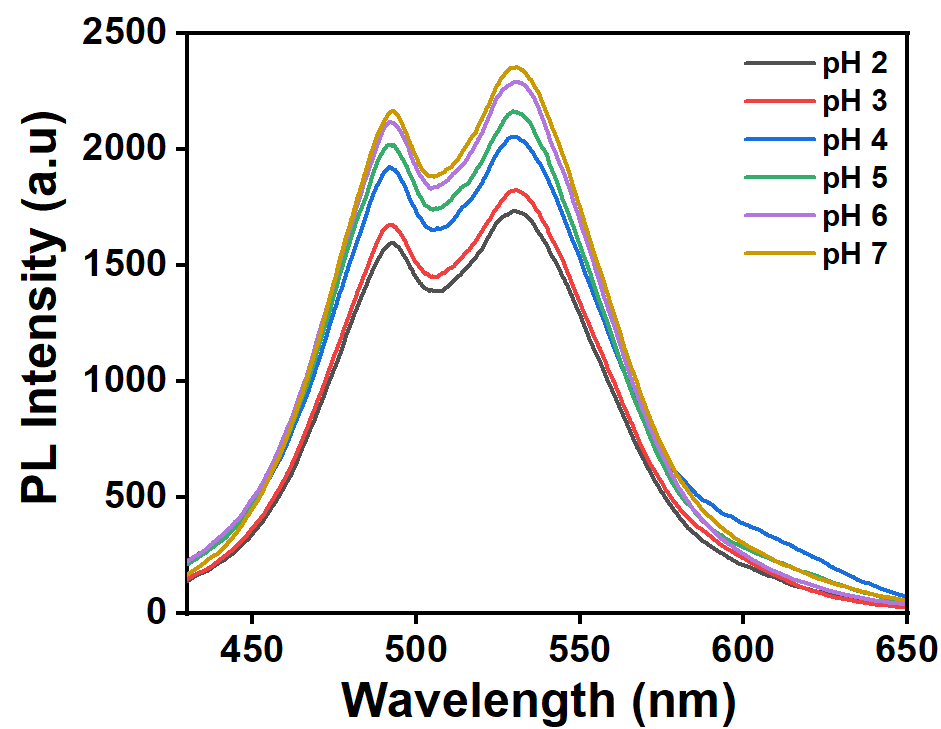
**

**Figure S7.** Emission spectra of BP-R microrobots in acidic pH (2-7).

**Limit of detection for Picric acid in neutral conditions**


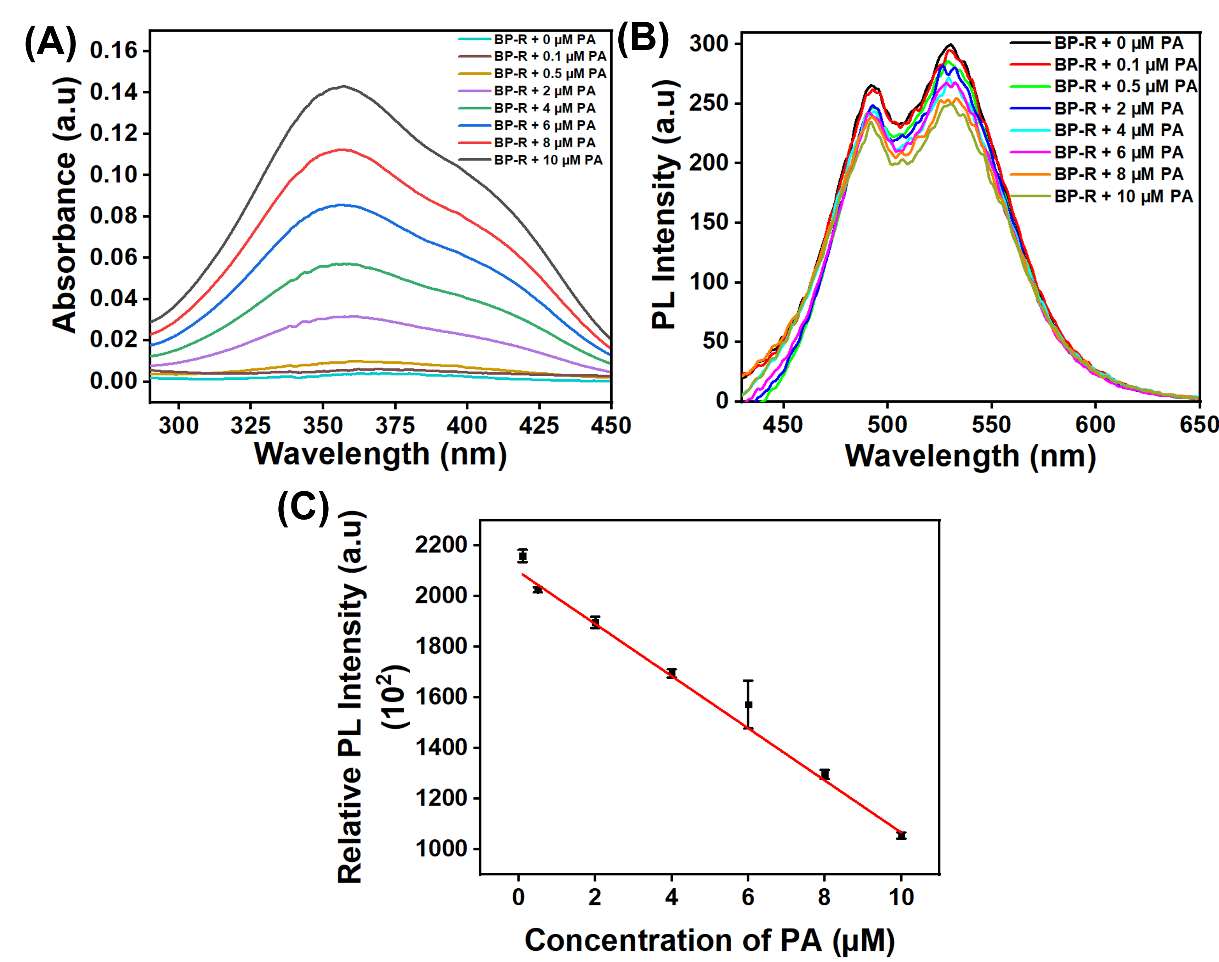


**Figure S8.** (A) Electronic absorption spectra, (B) emission spectra, (C) linear plot of PL emission intensity versus increasing concentration of picric acid (0.1 µM - 10 µM) at pH 7.

**Calculation of LOD (pH 7):**

Slope (k) = 1058.86 x 10^6^ intensity/M

Standard deviation (σ) = 76.55 intensity (n=6)

Limit of detection = 3.3 σ/k

= 3.3 x (76.55 /1058.86 x 10^6^) M

LOD = 0.238 x 10^-6^ M

= 238 nM.

**Calculation of LOD (pH 2):**

Slope (k) = 482.66 x 10^6^ intensity/M

Standard deviation (σ) = 37.41 intensity (n=6)

Limit of detection = 3.3 σ/k

= 3.3 x (37.41/482.66 x 10^6^) M

LOD = 0.255 x 10^-6^ M

= 255 nM.

**Electronic absorption spectra**

**
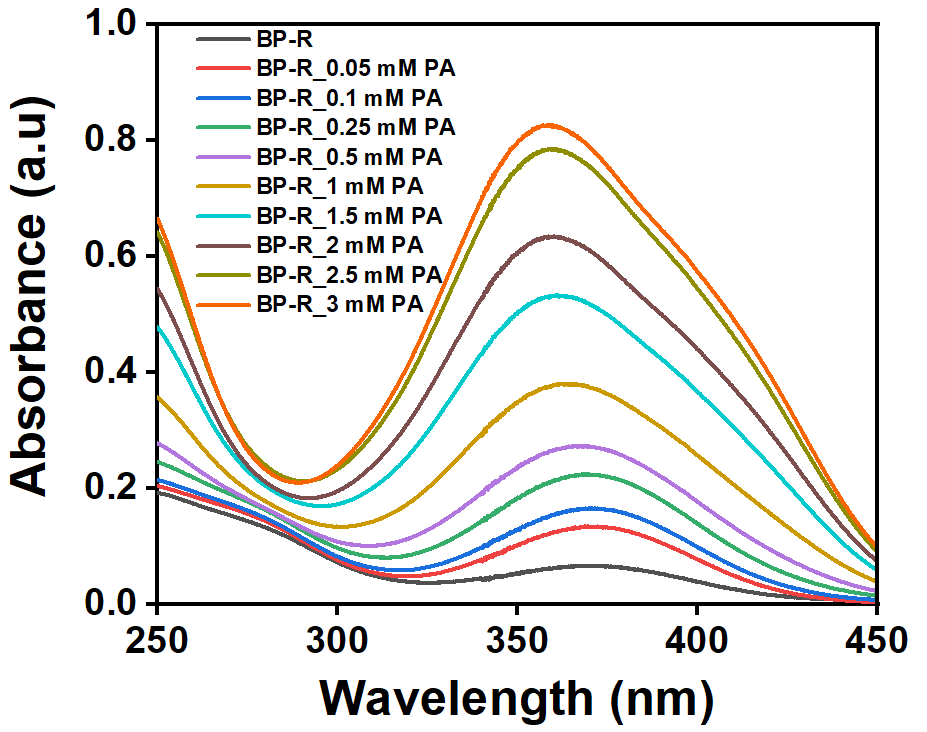
**

**Figure S9.** Electronic absorption spectra of BP-R microrobots with different concentrations of picric acid (0.1 - 3mM).

**Response time of microrobots**

**_
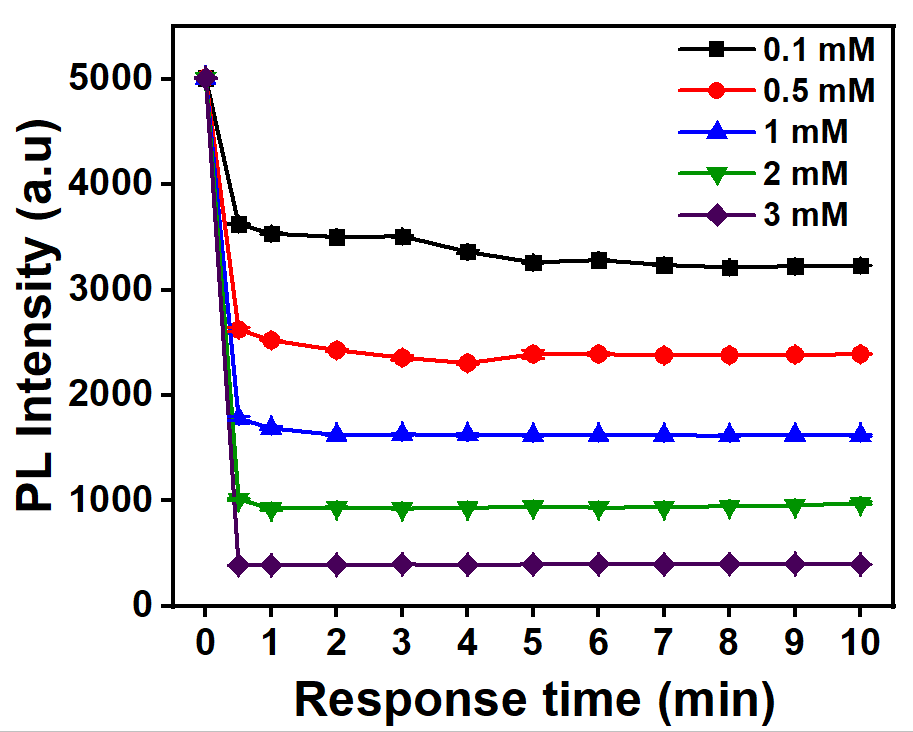
_**

**Figure S10.** Response time of BP-R microrobots toward different concentrations of picric acid (0.1, 0.5, 1, 2, and 3 mM).

**Effect of basic pH condition on the sensing of BP-R microrobots to picric acid**

**
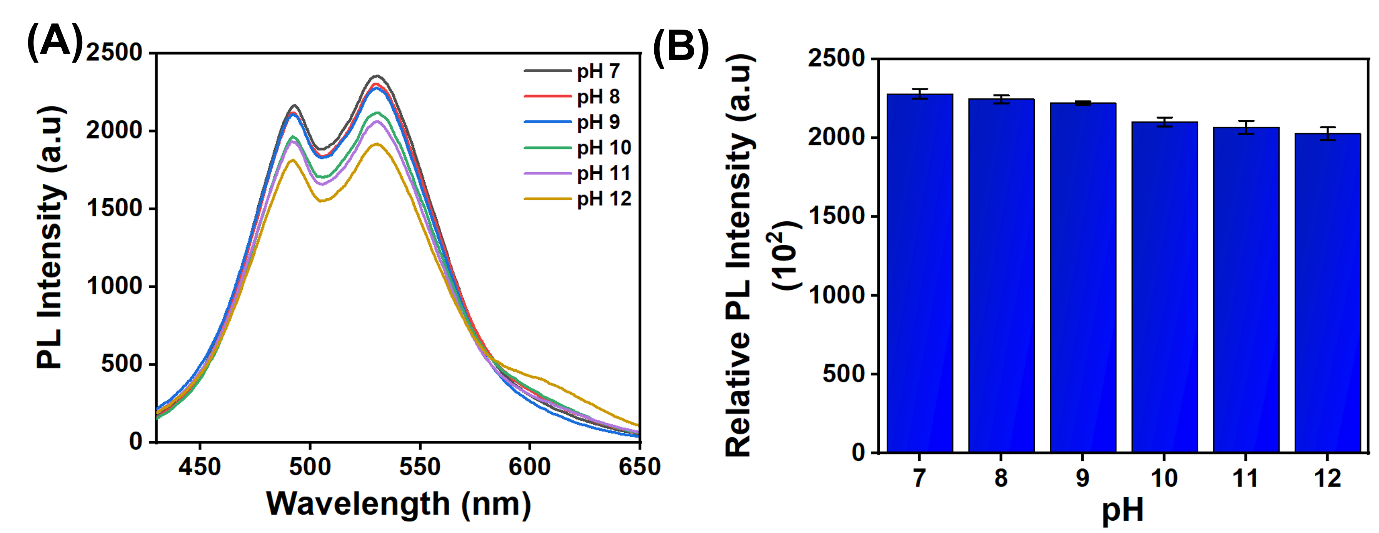
**

**Figure S11.** (A) Emission spectra, (B) relative emission intensity of BP-R microrobots in basic pH (7-12).

**Effect of metal ions on the sensing of BP-R microrobots to picric acid**

**
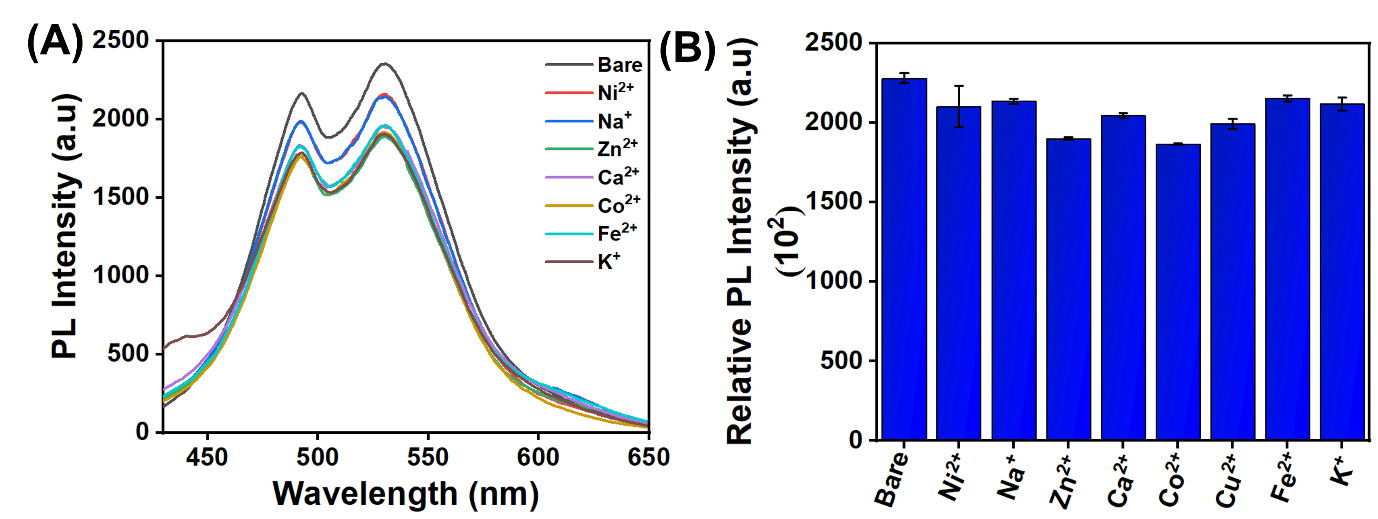
**

**Figure S12.** (A) Emission spectra, (B) relative emission intensity of BP-R microrobots on different metal ion species.

| Sr. No | Probes | Microrobot | Sensing in acidic medium | LOD (M) | Condition | Solvent system | Ref. |
| --- | --- | --- | --- | --- | --- | --- | --- |
| 1 | Nitrogen-Doped Carbon Dots | No | No | 33 × 10^− 9^ | Neutral | Aqueous | ^S1^ |
| 2 | Ni-MOF | No | No | 0.29 × 10^− 6^ | Neutral | Aqueous | ^S2^ |
| 3 | Cd(II) coordination polymer | No | No | 8.36 × 10^−6^ | Neutral | Aqueous | ^S3^ |
| 4 | Sr-tcbpe | No | No | 2.25 ×10^-6^ | Neutral | Ethanol | ^S4^ |
| 5 | Aniline-based covalent organic frameworks | No | No | 1.06 × 10^−9^ | Neutral | Aqueous | ^S5^ |
| 6 | N, S-doped carbon dots | No | No | 7.0×10^−8^ | Neutral | Aqueous | ^S6^ |
| 7 | Covalent Organic Cage | No | No | 2.7 × 10^− 9^ | Neutral | Aqueous | ^S7^ |
| 8 | Quinoline-based sensor | No | No | 9.33 × 10^− 6^ | Neutral | Aqueous | ^S8^ |
| 9 | Neutral red (NR) dye | No | No | 6.39×10^−7^ | Neutral | ACN | ^S9^ |
| 10 | Carbonized polymer dots | No | No | 318 x 10^-9^ | Neutral | Aqueous | ^S10^ |
| 11 | NiNCs@HS1 | No | No | 9.7×10^−7^ | Neutral | Aqueous | ^S11^ |
| 12 | Tryptanthrin-Spiropiperidine hybrid molecule (CTSP) | No | No | 5.03 × 10 ^−6^ | Neutral | DMSO/H_2_O (1:1) | ^S12^ |
| 13 | Porous organic polymer | No | No | 8 × 10^− 6^ | Neutral | Aqueous | ^S13^ |
| 14 | PAI | Yes | No | 214 × 10^− 9^ | Neutral | Aqueous | ^S14^ |
| 15 | BP-R | Yes | Yes | 238 × 10^− 9^  255 × 10^− 9^ | Neutral  Acidic | Aqueous | This Work |

**Comparison table for sensing performance of BP-R microrobots with other fluorescent probes to picric acid**

**Table S2.** Comparison of the sensing performance of BP-R microrobots with other fluorescent sensing probes to picric acid

**Reference:**

(S1) Mahto, M. Kr.; Samanta, D.; Shaw, M.; Shaik, M. A. S.; Basu, R.; Mondal, I.; Bhattacharya, A.; Pathak, A. Blue-Emissive Nitrogen-Doped Carbon Dots for Picric Acid Detection: Molecular Fluorescence Quenching Mechanism. *ACS Appl. Nano Mater.* **2023**, *6* (9), 8059–8070. https://doi.org/10.1021/acsanm.3c01523.

(S2) Chongdar, S.; Mondal, U.; Chakraborty, T.; Banerjee, P.; Bhaumik, A. A Ni-MOF as Fluorescent/Electrochemical Dual Probe for Ultrasensitive Detection of Picric Acid from Aqueous Media. *ACS Appl. Mater. Interfaces* **2023,** *15*, 14575−14586. https://doi.org/10.1021/acsami.3c00604.

(S3) Dou, L.; Tong, L.; Ma, C.-Y.; Dong, W.-K.; Ding, Y.-J. Inserting Auxiliary Ligand to Construct a Cd (II)-Based Salamo-like Coordination Polymer as Bifunctional Chemosensor for Detecting Picric Acid and S^2−^. *J. Mol. Struct.* **2023**, *1292*, 136162. https://doi.org/10.1016/j.molstruc.2023.136162.

(S4) Wang, C.; Zhang, X.-J.; Zhao, L.; Zhang, T.; Bai, F.-Y.; Sun, L.-X.; Xing, Y.-H. Multiple Stimulus Response Material Based on Sr-Tcbpe MOF for Mechanochromism, Visualization Labeling, and Etching Toward TNP. *ACS Appl. Mater. Interfaces* **2024**, *16* (34), 45214–45223. https://doi.org/10.1021/acsami.4c10799.

(S5) Wang, K.; Geng, T.-M.; Zhu, H.; Guo, C. The Preparation of the Flexible Aniline-Based Covalent Organic Frameworks Used for Uptaking Iodine and Sensing Picric Acid and Iodine. *Microporous and Mesoporous Mater.* **2024**, *363*, 112794 (1-10). https://doi.org/10.1016/j.micromeso.2023.112794.

(S6) Annamalai, K.; Ravichandran, R.; Annamalai, A.; Jeevarathinam, A.; Suresh, R.; Elumalai, S. Synthesis of Blue-Sparkling N, S-Doped Carbon Dots for Effective Detection of Nitro Explosive and Fe^3+^ Ion and Anti-Counterfeiting Studies. *Mater. Res. Bull.* **2025**, *181*, 113068. https://doi.org/10.1016/j.materresbull.2024.113068.

(S7) Mahto, A. K.; Barik, S.; Sarkar, M.; Madda, J. P. A Fluorescent Covalent Organic Cage for Ultrafast Detection of Picric Acid and HCl Vapor Sensing. *Chem. - Asian J.***2025**, *20* (3), e202400912 (1-10). https://doi.org/10.1002/asia.202400912.

(S8) Sudharsan, S.; Hemalatha, V.; Sarveswari, S.; Vijayakumar, V. A Highly Selective and Sensitive Quinoline-Based Fluorescent Turn-off Chemosensor for the Detection of Picric Acid. *J. Mol. Struct.* **2024**, *1317*, 139087 (1-11). https://doi.org/10.1016/j.molstruc.2024.139087.

(S9) Sarkar, P.; Tohora, N.; Mahato, M.; Ahamed, S.; Sultana, T.; Das, S. K. A Chromo-Fluorogenic Probe for Selective Detection of Picric Acid Alongside Its Recovery by Aliphatic Amines and Construction of Molecular Logic Gates. *J. Fluoresc.* **2023**, *35* (2), 751–767. https://doi.org/10.1007/s10895-023-03555-y.

(S10) Qiao, R.; Li, Y.; Zhu, R.; Bai, H.; Zhao, C.; Zu, B.; Cai, Z. Carbonized Polymer Dots for Discrimination of 2,4,6-Trinitrotoluene and 2,4,6-Trinitrophenol. *J. Hazard. Mater.* **2025**, *491*, 137944. https://doi.org/10.1016/j.jhazmat.2025.137944.

(S11) Jain, N.; Kaur, N. Construction of 1,8-Naphthalimide Modified Nickel Nanoclusters Based Nanohybrid Platform for the Detection of Fast Green and Picric Acid. *Food Chem.* **2025**, *485*, 144546. https://doi.org/10.1016/j.foodchem.2025.144546.

(S12) Al-Sharifi, H. K. R.; Krishnan, D.; Jayasree, E. G.; Deepthi, A. Tryptanthrin Incorporated Spiropiperidine Derivative as a Fluorescent Chemosensor for Picric Acid Detection. *Spectrochim. Acta - A: Mol. Biomol. Spectrosc.* **2025**, *339*, 126311. https://doi.org/10.1016/j.saa.2025.126311.

(S13) Mondal, B.; Das, G. Dual Functional Porous Organic Polymer: Reversible Iodine Capture and Selective Sensing of Picric Acid. *React. Funct. Polym.* **2024**, *194*, 105800 (1-8). https://doi.org/10.1016/j.reactfunctpolym.2023.105800.

(S14) Senthilnathan, N.; Oral, C. M.; Pumera, M. Magneto-Fluorescent Microrobots with Selective Detection Intelligence for High-Energy Explosives and Antibiotics in Aqueous Environments. *ACS Appl. Mater. Interfaces* **2025**, *17* (14), 21691–21704. https://doi.org/10.1021/acsami.5c02259.
